# Fibrillar adhesives with unprecedented adhesion strength, switchability and scalability

**DOI:** 10.1093/nsr/nwae106

**Published:** 2024-03-20

**Authors:** Changhong Linghu, Yangchengyi Liu, Xudong Yang, Dong Li, Yee Yuan Tan, Mohamed Haziq Bin Mohamed Hafiz, Mohammad Fadhli Bin Rohani, Zihao Du, Jiangtao Su, Yan Li, Yucheng Huo, Hanyan Xu, Xiufeng Wang, Yifan Wang, Jing Yu, Huajian Gao, K Jimmy Hsia

**Affiliations:** School of Mechanical and Aerospace Engineering, Nanyang Technological University, Singapore 639798, Singapore; School of Mechanical and Aerospace Engineering, Nanyang Technological University, Singapore 639798, Singapore; School of Materials Science and Engineering, Xiangtan University, Xiangtan 411105, China; School of Mechanical and Aerospace Engineering, Nanyang Technological University, Singapore 639798, Singapore; School of Mechanical and Aerospace Engineering, Nanyang Technological University, Singapore 639798, Singapore; School of Mechanical and Aerospace Engineering, Nanyang Technological University, Singapore 639798, Singapore; School of Mechanical and Aerospace Engineering, Nanyang Technological University, Singapore 639798, Singapore; School of Mechanical and Aerospace Engineering, Nanyang Technological University, Singapore 639798, Singapore; School of Mechanical and Aerospace Engineering, Nanyang Technological University, Singapore 639798, Singapore; Department of Engineering Mechanics, Zhejiang University, Hangzhou 310027, China; School of Materials Science and Engineering, Nanyang Technological University, Singapore 639798, Singapore; School of Mechanical and Aerospace Engineering, Nanyang Technological University, Singapore 639798, Singapore; School of Mechanical and Aerospace Engineering, Nanyang Technological University, Singapore 639798, Singapore; School of Materials Science and Engineering, Nanyang Technological University, Singapore 639798, Singapore; School of Materials Science and Engineering, Xiangtan University, Xiangtan 411105, China; School of Mechanical and Aerospace Engineering, Nanyang Technological University, Singapore 639798, Singapore; School of Materials Science and Engineering, Nanyang Technological University, Singapore 639798, Singapore; School of Mechanical and Aerospace Engineering, Nanyang Technological University, Singapore 639798, Singapore; Institute of High-Performance Computing, A*STAR, Singapore 138632, Singapore; Mechano-X Institute, Applied Mechanics Laboratory, Department of Engineering Mechanics, Tsinghua University, Beijing 100084, China; School of Mechanical and Aerospace Engineering, Nanyang Technological University, Singapore 639798, Singapore; School of Chemistry, Chemical Engineering and Biotechnology, Nanyang Technological University, Singapore 639798, Singapore

**Keywords:** JKR-DMT transition, rubber-to-glass (R2G) transition, R2G fibrillar adhesives, shape memory polymers, adhesion switchability and scalability

## Abstract

Bio-inspired fibrillar adhesives have received worldwide attention but their potentials have been limited by a trade-off between adhesion strength and adhesion switchability, and a size scale effect that restricts the fibrils to micro/nanoscales. Here, we report a class of adhesive fibrils that achieve unprecedented adhesion strength (∼2 MPa), switchability (∼2000), and scalability (up to millimeter-scale at the single fibril level), by leveraging the rubber-to-glass (R2G) transition in shape memory polymers (SMPs). Moreover, R2G SMP fibrillar adhesive arrays exhibit a switchability of >1000 (with the aid of controlled buckling) and an adhesion efficiency of 57.8%, with apparent contact area scalable to 1000 mm^2^, outperforming existing fibrillar adhesives. We further demonstrate that the SMP fibrillar adhesives can be used as soft grippers and reusable superglue devices that are capable of holding and releasing heavy objects >2000 times of their own weight. These findings represent significant advances in smart fibrillar adhesives for numerous applications, especially those involving high-payload scenarios.

## Teaser

New design paradigm by harnessing the JKR-DMT transition in shape memory polymers leads to fibrillar adhesives that possess exceptionally strong, switchable, and scalable adhesion for high-payload applications.

## INTRODUCTION

Smart adhesives, which provide strong adhesion when needed and easy detachment when desired, are extensively utilized across a broad spectrum of applications, ranging from agriculture to various industrial domains [1–6]. These adhesives inspired by natural fibrillar adhesive systems [1,7–10], which relies on ubiquitous Van der Waals interactions rather than chemical interactions, are adaptable to various substrates and are more sustainable, and have shown promising potentials in soft grippers [3,4,11], robotics [4,6,8,12–15], advanced manufacturing [2,6,16–20], and wearables [1,21,22]. However, existing approaches to achieving switchable adhesion using fibrils, based on mechanical principles such as controlled shearing [23], buckling [24], or peeling [4,25], have been limited by the trade-off between adhesion strength and switchability (the ratio of the maximum adhesion load to the lowest detachment load) [26], as well as the scaling limits at both the single fibril [1] and fibrillar array levels [1,4] (Sections S1–S3).

Theoretical models have revealed [27–29] that the adhesion strength of fibrils is strongly dependent on their size at the single fibril level. Small fibrils can achieve attachment in the DMT-like regime [30,31], reaching the theoretical adhesion strength ${{{\boldsymbol{\sigma }}}_{{\mathrm{th}}}}$ and leading to size-independent strong adhesion with the fibril detaching as a whole. In nature, adhesive fibrils that approach the DMT-like regime are very small, ranging from a few hundred nanometers to a few micrometers [1,27]. On the other hand, large fibrils tend to detach in the JKR-like regime [31,32], resulting in weak adhesion due to stress concentration at the edge [1] with the fibril detaching from the edges. As the radius of a single fibril increases from 100 $\mu {\mathrm{m}}$ to 1 mm, the adhesion strength decreases from ∼100 kPa to <25 kPa, imposing limitations on the size and complexity of fibril geometry required to achieve strong adhesion [1,33], and leading to various practical challenges such as expensive micro/nano-fabrication processes [1], susceptibility to lateral collapse, bundling [1], fracture and abrasion [13]. Despite various proposed optimizations (e.g. modulus gradients [1,13], mushroom-shaped tips [1,4,17,23,33,34]), the adhesion strength of mm-scale fibrils remains significantly lower than the intrinsic level of Van der Waals interactions of MPa, and achieving both high adhesion strength and easy detachment remains challenging [2].

At the array level, the effective adhesion strength ${{{\boldsymbol{\sigma }}}_{{\mathrm{eff}}}}$ (the adhesion force over the apparent contact area) may not scale up with the number of fibrils due to uneven load sharing [1] among fibrils. This uneven load sharing can be caused by interfibrillar mechanical interactions and deformation of the backing layer [1,4]. Increasing the stiffness of the backing layer can improve adhesion efficiency (defined as the ratio of the effective adhesion strength ${{{\boldsymbol{\sigma }}}_{{\mathrm{eff}}}}$ of the fibril arrays under a certain apparent contact area to the theoretical adhesion strength ${{{\boldsymbol{\sigma }}}_{{\mathrm{th}}}}$) but reduces adhesive system compliance during contact [3,35], limiting its adaptability to different surface textures. Some designs have utilized a releasable stiffer backing [4] to address this issue, but only a limited percentage (<26%) [4] of the adhesive fibrils’ full potential can be exploited when scaling up the apparent contact area.

**Figure 1.**
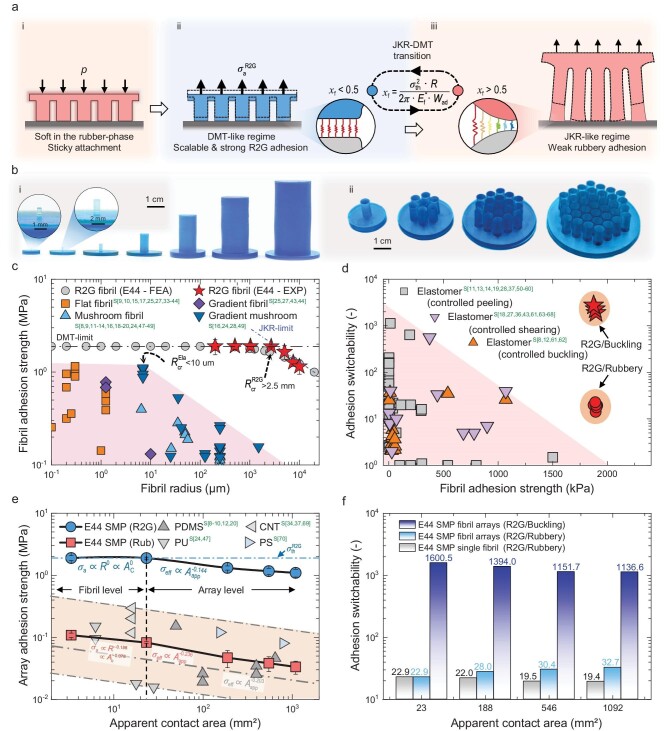
Overview of the R2G fibrillar adhesives. (a) Illustrations showing the working principle of the R2G phase changing fibrillar adhesive. (i) On-demand attachment in the rubber-phase. During the contact process, the adhesive fibrils become soft upon transitioning into the rubber-phase under stimulation, which facilitates sticky attachment of the adhesive fibrils on the adherend when Dahlquist criterion (i.e. *E* <1 MPa) is satisfied. (ii) Strong and scalable adhesion in the DMT-like regime. After attachment, the adhesive fibrils become stiff when transitioning into the glass-phase upon reverse stimulation. When loaded in the stiff-state, the adhesive fibrils work under the DMT-like regime with uniform stress distribution at every fibril-adherend contact interface when χ*_f_* <0.5 and equal load sharing among different fibrils, resulting in strong and scalable adhesion to support heavy loads. (iii) On-demand detachment in the JKR-like regime. The adhesive fibrils become soft upon transitioning into the rubber-phase under restimulation. When loaded in the soft-state, the adhesive fibrils work under the JKR-like regime with high stress concentration at the periphery of every fibril-adherend interface when χ*_f_* >0.5 and uneven load sharing among different fibrils as well as progressive detachment from the periphery inwards, resulting in weak adhesion for easy detachment. (b) Photos of (i) single adhesive fibrils with different fibril radii and (ii) fibril arrays with different apparent contact areas made of E44-SMP. (c) Adhesion strength scaling performance of the R2G adhesive fibrils made of E44-SMP at the single fibril level compared to those made of conventional elastomers. The R2G adhesive fibrils made of E44-SMP can sustain the DMT-like strong adhesion with fibril radius scaled all the way up to the millimeter-size, breaking through the scaling limit of elastomeric adhesives. (d) Adhesion switchability versus adhesion strength of the R2G adhesive fibrils made of E44-SMP as compared to those made of elastomers actuated by controlled peeling (gray squares), controlled buckling (orange upper triangles), and controlled shearing (purple lower triangles). (e) Adhesion strength scaling performance of the R2G fibrillar adhesives made of E44-SMP at the array level compared to fibrillar adhesives made of elastomers. The SMP fibrillar adhesive showed an improved adhesion efficiency (57.8%) over the elastomeric ones even when the apparent contact area is scaled up to 1000 mm^2^. (f) Array adhesion switchability of the R2G fibril arrays and the single fibril adhesive made of E44-SMP. The switchability can be increased by two orders of magnitude through controlled buckling in R2G fibril arrays. Details and origin of these literature data for elastomers are given in Section S1 and S2. ‘S [x]’ in c, d and e refers to the references in the online Supplementary Materials.

To overcome these limitations, we propose a new design paradigm that leverages the JKR-DMT transition of adhesive fibrils’ adhesion regime. This design enables the development of strong, switchable, and scalable smart fibrillar adhesives by tuning geometrical, elastic, and adhesive parameters, as depicted in Fig. 1a. We introduce the Rubber-to-Glass (R2G) phase changing fibrillar adhesives concept, utilizing smart materials with two distinct phases: a soft rubber-phase and a stiff glass-phase [36,37]. The adhesive transitions between these two phases achieve the desired adhesion regimes. During attachment (Fig. 1a–i), the R2G fibrillar adhesive is in the soft rubber-phase, facilitating enhanced contact and sticky attachment [38]. Upon transitioning to the glass-phase after contact, the fibrillar adhesive exhibits strong R2G adhesion (contact in the rubber-phase and detach in the glass-phase) [29], with uniform stress distribution at each fibril and equal load sharing among fibrils (Fig. 1a–ii). During detachment, the fibrillar adhesive returns to the soft rubber-phase, which induces detachment in the JKR-like regime at each fibril and uneven load sharing among fibrils (Fig. 1a–iii), resulting in weak rubber-phase adhesion for easy detachment.

To demonstrate the capabilities of the R2G fibrillar adhesive, we fabricated adhesive fibrils with different radii (Fig. 1b-i) and fibril arrays with varying apparent contact areas (Fig. 1b-ii) using a thermally-controlled E44 shape-memory polymer (E44-SMP) [36].

## RESULTS AND DISCUSSION

### Adhesion scaling of individual fibrils

Figure 1c presents a summary of the adhesion scaling performance of E44-SMP R2G adhesive fibrils (Fig. 1b–i) compared to those made of other elastomers (Section S1). In conventional elastomeric adhesive fibrils, the maximum radius for the DMT-like regime, ${\boldsymbol{R}}_{{\mathrm{cr}}}^{{\mathrm{Ela}}}$, is ∼10 $\mu {\mathrm{m}}$. In contrast, E44-SMP R2G adhesive fibrils can maintain the DMT-like regime when its radius is as large as ${\boldsymbol{R}}_{{\mathrm{cr}}}^{{\mathrm{R}}2{\mathrm{G}}}\ $∼2.5 mm, as demonstrated by experiments and confirmed by finite element (FEA) simulations (Fig. 1c). E44-SMP R2G adhesive fibrils can achieve adhesion strength of 1.893 MPa with radius up to the millimeter-size.

Figure 1d (and Fig. S2) shows the trade-off between adhesion strength and switchability. For conventional elastomeric adhesive fibrils, increasing the adhesion strength to ∼2 MPa significantly reduces the adhesion switchability to ∼1, making the adhesion nearly non-switchable. In contrast, E44-SMP R2G adhesive fibrils, under R2G conditions, achieve adhesion switchability of 20 (red circles in Fig. 1d) due to JKR-like detachment in the rubber-phase. By subjecting R2G adhesive fibrils to a large preload in the rubber-phase to induce fibril buckling, the detachment strength can be further reduced by two orders of magnitude, leading to an extraordinary adhesion switchability of approximately 2000 (red stars in Fig. 1d). Consequently, the R2G adhesive fibrils can achieve unprecedented adhesion strength and switchability.

### Adhesion scaling of fibril arrays

To fully benefit DMT-like adhesion, the radius of an individual E44-SMP R2G adhesive fibril cannot exceed ${\boldsymbol{R}}_{{\mathrm{cr}}}^{{\mathrm{R}}2{\mathrm{G}}}$ (Fig. 1c). Devices requiring higher load capacity can employ fibril arrays with radius of each fibril smaller than ${\boldsymbol{R}}_{{\mathrm{cr}}}^{{\mathrm{R}}2{\mathrm{G}}}$ (Section S9). The adhesion load capacity of a fibril array device scales with its apparent contact area. Our experimental measurements show that, for a device with the apparent contact area of 1000 mm^2^, the load capability of R2G fibril arrays is 1.4 times that of the R2G single fibril and 23.89 times that of elastomeric fibril arrays (Fig. S10).

Figure 1e shows the measured scaling performance of the effective adhesion strength of fibril arrays compared to the elastomeric ones. The measured rubber-phase array adhesion strength of the SMP fibril arrays follows a scaling law ${\boldsymbol{\sigma }}_{{\mathrm{eff}}}^{{\mathrm{Rub}}} = {\boldsymbol{A}}_{{\mathrm{app}}}^{ - 0.236}$, similar to the scaling law ${\boldsymbol{\sigma }}_{{\mathrm{eff}}}^{{\mathrm{Ela}}} = {\boldsymbol{A}}_{{\mathrm{app}}}^{ - 0.203}$ observed for elastomeric ones, where ${{{\boldsymbol{A}}}_{{\mathrm{app}}}}$ is the apparent contact area. In contrast, R2G fibril arrays show a much slower decay rate of ${{{\boldsymbol{\sigma }}}_{{\mathrm{eff }} }}={\boldsymbol{A}}_{{\mathrm{app}}}^{ - 0.144}$, resulting in an adhesion efficiency of 57.8% when the apparent contact area is scaled up to 1000 mm^2^. The R2G fibrillar adhesive can thus utilize more than half of its full potential, representing a significant improvement over elastomeric adhesives, even when compared to the state-of-the-art result (26%) achieved through complex controlled-load-sharing designs [4].

Dividing a single fibril into fibril arrays can further enhance adhesion switchability, as demonstrated by the experimental results in Fig. 1f. Through switching between the R2G condition and rubber-phase, single adhesive fibrils have intermediate switchability (∼20) that decreases with increasing contact area. After dividing into fibril arrays with each fibril radius of 2.5 mm, the switchability increases slightly with the contact area. Preload-induced buckling of fibril arrays can reduce the rubber-phase detachment strength, leading to a dramatic increase in switchability (>1000).

### Materials' selection and characterization

**Figure 2.**
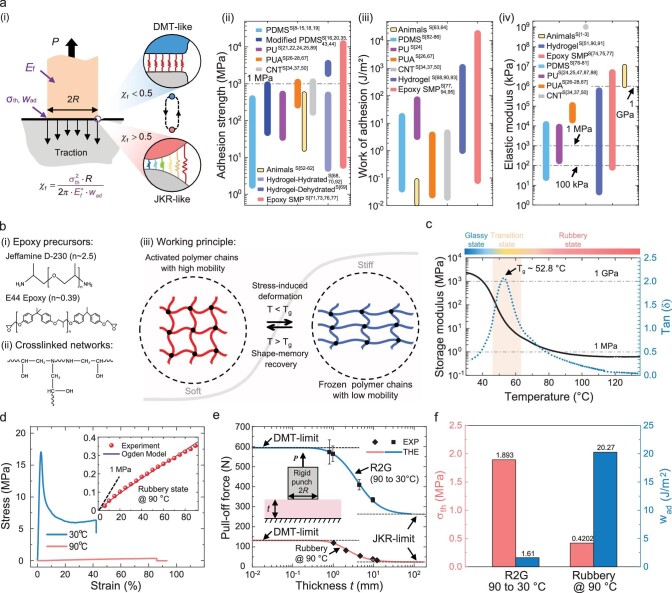
Materials of the R2G fibrillar adhesive. (a) Key material parameters governing the adhesion regime of an adhesive fibril. (i) Illustrations showing the mechanics model and key parameters determining the transition between JKR-like and DMT-like adhesion regimes of an adhesive fibril on a rigid substrate. Summary and comparison of the (ii) adhesion strength, (iii) work of adhesion and (iv) elastic modulus values of typical materials used for the fibrillar adhesives. (Details and origin of the literature data for elastomers are given in Section S1 and S2. ‘S [x]’ means the references in the Supplementary Materials). (b) Molecular structures of the E44-SMP (i) precursors and (ii) crosslinked networks, and (iii) the illustration of related working principle. (c–d) Mechanical behaviors of the E44 epoxy SMP. (c) Storage modulus of the E44-SMP under different temperatures obtained from DMA tests. (d) Typical strain-stress curves of the E44-SMP in the glass-phase (30°C) and rubber-phase (90°C), at the tension speed 10 mm/min. Inset shows the hyperelastic fitting of the rubber-phase strain-stress curves using the Ogden model. (e–f) Adhesive parameters of the E44-SMP. (e) Pull-off forces measured on the E44-SMP samples with different thicknesses utilizing a rigid glass punch (20 mm in diameter). (f) Fitted adhesive parameters (theoretical adhesion strength and work of adhesion) between the E44-SMP and the glass punch from the measured pull-off forces in (e).

The working principle depicted in Fig. 1a and Fig. 2a–i requires that the materials for R2G adhesive fibrils must satisfy: (1) sufficient stickiness (elastic modulus ***E***_f_ <1 MPa) in the rubber-phase to adhere to rough surfaces according to the Dahlquist criterion [38]; (2) transition between JKR-DMT adhesion regimes occurs in fibrils of millimeter size. Theoretical studies [28,31] indicate that this transition depends on geometric, elastic, and interfacial properties of the adhesive system. The selection of materials and the design of fibrillar structures are essential for the proposed R2G adhesive system to exhibit distinctive, easily achievable JKR/DMT-like regimes. Elastic modulus ***E***_f_, theoretical adhesion strength **σ**_th_ and work of adhesion ${{{\boldsymbol{w}}}_{ad}}$ are key parameters affecting the JKR-DMT transition. The underlying physics for this transition is the ratio between the cohesive zone length *l*_cz_ and the fibril radius *R*. The adhesion regime is DMT-like if *l*_cz_ >*R* and JKR-like otherwise. Specifically, the JKR-DMT transition of an elastomeric fibril on a rigid substrate is governed by the dimensionless parameter [28]


(1) \begin{eqnarray*}
{{{{\bf \chi }}}_{{\mathrm{f\ }}}}{\mathrm{\ }} = \frac{{{{\bf \sigma }}_{{\mathrm{th}}}^2 \cdot {\boldsymbol{R}}}}{{2{\mathrm{\pi }} \cdot {\boldsymbol{E}}_{\mathrm{f}}^* \cdot {{{\boldsymbol{w}}}_{ad}}}},
\end{eqnarray*}

where ***R*** is the radius of the fibril and ${\boldsymbol{E}}_{\mathrm{f}}^* = \ {{{\boldsymbol{E}}}_{\mathrm{f}}}/( {1 - {{v}^2}} )$, ***E***_f_ and *v* being the Young's modulus and Poisson's ratio of the fibril, respectively. The adhesion regime is DMT-like when ${{{\mathrm{\chi }}}_{{\mathrm{f\ }}}} < 0.5$ and JKR-like for ${{{\mathrm{\chi }}}_{{\mathrm{f\ }}}} > 0.5$. To ensure that the adhesive fibril remains in the DMT-like regime to support high load (Fig. 1a–ii and Fig. 2a–i), ${{{\mathrm{\chi }}}_{{\mathrm{f\ }}}}$ must be smaller than 0.5; and to detach easily in the JKR-like regime (Fig. 1a–iii and Fig. 2a–i), ${{{\mathrm{\chi }}}_{{\mathrm{f\ }}}}$ must be larger than 0.5 during operation.

Figure 2a–ii to iv provide the range of elastic modulus ***E***, adhesion strength ${{{\boldsymbol{\sigma }}}_a}$, and work of adhesion ${{{\boldsymbol{w}}}_{ad}}$ of typical materials for fibrillar adhesives on glass substrates (see details in Section S4). Among them, animal adhesive fibrils [27] and carbon nanotube (CNT) [39] fibrils are stiff (>1 GPa) with a low ${{{\boldsymbol{w}}}_{ad}}$ (∼10 mJ/m^2^), exhibiting a DMT-like regime for small fibril sizes. Other synthetic materials, such as polydimethylsiloxane (PDMS), are relatively soft, with large ${{{\boldsymbol{w}}}_{ad}}$ (∼0.1–10 J/m^2^). The adhesion strength of these synthetic fibrils is typically below 1 MPa, and achieving the DMT-like regime is challenging [1] unless the fibril tip is optimized (e.g. using mushroom-shaped tips [17,33]) at micro-scales (<10 µm). In contrast, smart materials such as hydrogels [40,41] and SMPs [3,36,37] offer a wide range of ***E***, ${{{\boldsymbol{\sigma }}}_a}$, and ${{{\boldsymbol{w}}}_{ad}}$ values. However, hydrogels are not suitable for long-term applications due to dehydration over time. SMPs [3,36,37], in contrast, are good candidates due to their good material stability, widely tunable modulus (∼10 kPa–10 GPa) during R2G transition, and strong adhesion to various materials (∼MPa).

Indeed, SMPs have been employed as smart adhesives for over a decade and a half, utilizing their tunable modulus effect [42] or shape-memory effect [43]. However, existing reports predominantly focus on technological aspects and are based on conventional mechanics models for elastic bodies. They fall short of explaining the underlying mechanisms of notable adhesion enhancement and unique preload dependence. Recent advancements in the contact mechanics of R2G adhesion [26,29] have revealed the pivotal role of shape-locking during adhesion, a factor previously underappreciated. Importantly, most studies showcasing high adhesion strength and switchability in SMPs, based on conventional elasticity theory, incorporate micro-structures, and none addressed the scalability challenges of smart adhesives at the fibril and array levels—an ongoing limitation for bio-inspired fibrillar adhesives. Within this context, our proposed breakthrough adhesion design paradigm, anchored in recent fundamental advancements in R2G adhesion [26,29], demonstrates unmatched adhesion strength, switchability, and scalability.

To demonstrate the feasibility of our adhesive paradigm, we selected a thermally tunable E44-SMP [36] (Section S5) as the adhesive material that exhibits R2G transition (Fig. 2b). It should be noted that, while we employ E44-SMP as an illustrative example of our adhesion design paradigm, the applicability of this paradigm extends beyond the E44-SMP or SMPs in general. This design concept, which leverages the transition between JKR-DMT adhesion regimes via phase changes, can also be realized using other R2G-type materials, such as liquid metals [44], liquid crystal polymers [45], or even jamming materials [46].

The highly tunable modulus of E44-SMP, ranging from 2 GPa at room temperature (25–30°C) to 1 MPa at 90°C, satisfies the Dahlquist criterion for stickiness, as shown in Fig. 2c. Figure 2d presents typical stress-strain curves of the E44-SMP at different temperatures. In the glass-phase (30°C), it exhibits a linear elastic behaviour; whereas in the rubber-phase (90°C), it shows a hyperelastic behaviur that can be characterized by the first-order Ogden–Roxburgh model (Section S5). The **σ_th_** and ${{{\boldsymbol{w}}}_{ad}}$ are the two other key parameters governing the JKR-DMT transition. Pull-off tests (Section S6) were conducted using flat punches on SMP samples of various thicknesses, as illustrated in Fig. 2e. By fitting the measured results to the theoretical expression [31] relating the pull-off force *P*_c_ and the sample thickness, we can determine the values of **σ_th_** and ${{{\boldsymbol{w}}}_{ad}}$, as shown in Fig. 2f. The results show that, under R2G conditions (contact at 90°C, detach at 30°C), E44-SMP demonstrates a high adhesion strength of 1.893 MPa compared to the rubber-phase (90°C contact/detach) strength of 0.4202 MPa. However, the work of adhesion in the glass-phase (1.61 J/m^2^) is lower than that in the rubber-phase (20.27 J/m^2^). These measured ***E*_f_, σ_th_** and ${{{\boldsymbol{w}}}_{ad}}$ values will be used in designing the R2G smart fibrillar adhesives.

### Viable parameter space for JKR-DMT transition and validation

**Figure 3.**
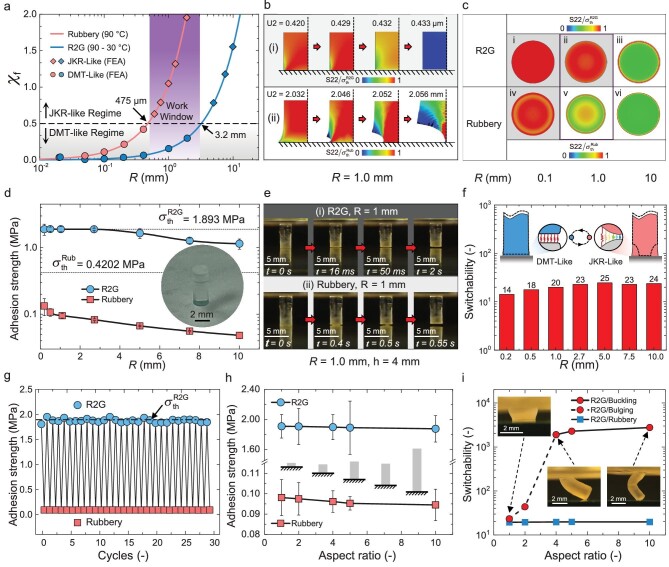
Design, validation, and characterization of the R2G fibrillar adhesive at the fibril level using E44-SMP. (a) Theoretical predictions and FEA validations of the adhesion regimes of the SMP adhesive fibril under various radii. The purple shadowed ‘work window’ indicates the range of the fibril radius within which the R2G and rubber-phase adhesion regimes of a single fibril are DMT-like and JKR-like, respectively. (b) FEA simulation results of the detachment processes of the SMP adhesive fibril in the work window (*R* = 1 mm) under the (i) R2G condition and (ii) rubber-phase. (c) Normal stress distribution at the SMP adhesive fibril–glass substrate interface with various fibril radii under the (i–iii) R2G condition and (iv–vi) rubber-phase. (d) Measured adhesion strength of an individual SMP adhesive fibril with a glass substrate under different radii. Inset shows the photo of an SMP adhesive fibril (*R* = 1 mm, *h* = 4 mm). (e) Snapshots of the detachment process of an individual SMP adhesive fibril (*R* = 1 mm, *h* = 4 mm) under the (i) R2G condition and (ii) rubber-phase. (f) Switchability of an individual SMP adhesive fibril under different radii. (g) Measured adhesion strength of the SMP adhesive fibril during 30 repeated cycles on a glass adherend. (h) Measured adhesion strength of an individual SMP adhesive fibril (*R* = 1 mm) under the R2G condition and rubber-phase with various aspect ratios. (i) Influence of the aspect ratio on the switchability of the SMP adhesive fibrils (*R* = 1 mm). Insets show the configurations of the deformed SMP fibril under a large preload.

The transition between adhesion regimes of an elastomeric fibril on a rigid substrate is governed by Eq. (1), which helps determine the size of fibril radius where the adhesion regime switches from the DMT-like regime under R2G condition to the JKR-like regime in the rubber-phase. The solid lines and the circular/diamond dots in Fig. 3a represent the theoretical predictions and FEM simulation results, respectively, of the transition between DMT-like and JKR-like regimes under R2G conditions (blue) and in the rubber-phase (pink). Taking ${{{{\bf \chi }}}_{{\mathrm{f\ }}}} = 0.5$ as the critical value at which the transition occurs, the results indicate that the fibril radius *R* must be smaller than 3.2 mm to maintain the DMT regime under R2G conditions, and larger than 475 *μ*m to reach JKR regime in the rubber-phase, suggesting a viable fibril size range of $475{\mathrm{\ }}\mu {\mathrm{m}} < R < 3.2{\mathrm{\ mm}}$ for strong adhesion and easy detachment. Large fibrils (*R* >3.2 mm) will result in a decrease in adhesion strength due to their JKR-like detachment. Conversely, small fibrils (*R* <475 *μ*m) will remain in the DMT-like regime, resulting in limited adhesion switchability, as demonstrated in previous studies [47].

The criterion based on ${{{{\bf \chi }}}_{{\mathrm{f\ }}}}$assumes infinite fibril height $( {h \gg R} )$ and material incompressibility $( {v = 0.5} )$. To assess more accurately the adhesion regime transition with finite fibril height and different Poisson's ratio, we conducted FEA simulations (ABAQUS 2022) with a cohesive zone model (Section S7). The results show that, under the R2G condition, the adhesion regime transition occurs at a fibril radius of $3{\mathrm{\ mm}}$ and, in the rubber-phase, it occurs at $0.4{\mathrm{\ mm}}$. Figure 3b–i and ii show typical detachment processes using FEA simulations in the DMT-like and JKR-like regimes. In the DMT-like regime (Fig. 3b–i, $R = 1{\mathrm{\ mm}},$ R2G condition), the fibril detaches uniformly at the contact interface, while in the JKR-like regime (Fig. 3b–ii, $R = 1{\mathrm{\ mm}},$ rubber-phase), detachment initiates at the periphery and propagates inwards. Figure 3c–i, ii, iii (R2G condition) and 3c–iv, v, vi (rubber-phase) show normal stress distributions at the contact interface for fibril radii of 0.1, 1.0 and 10 mm, with uniform stress in the DMT-like regime (Fig. 3c–i, ii, iv) and highly concentrated stress at the edge in the JKR-like regime (Fig. 3c–iii, v, vii), resulting in strong and weak adhesion, respectively. The FEA simulations validate the theoretical prediction of the JKR-DMT transition using the criterion ${{{{\bf \chi }}}_{{\mathrm{f\ }}}}$ for the E44-SMP R2G adhesive fibrils, suggesting a ‘work window’ of radius ranging from 0.5–3.0 mm to achieve DMT-like adhesion under R2G condition and JKR-like detachment in the rubber-phase. Although the work window shown here is based on the chosen temperatures (30°C for the glass-phase and 90°C for the rubber-phase), the design rationale provided by Eq. (1) can also account for its impact on the modulus and adhesive parameters [48], as discussed in our previous study [29].

### Characterization of R2G adhesive fibrils

To validate the parameter space for viable R2G adhesive fibrils, we fabricated single fibrils with different radii *R* and an aspect ratio $h/R = 4$ (Fig. 1b–i) using molding methods [3]. Pull-off tests on E44-SMP fibrils of various radii under R2G conditions (Section S8) were conducted to measure their adhesion strength, as shown in Fig. 3d. The measured adhesion strength matches the theoretical value $( {1.893{\mathrm{\ MPa}}} )$ for radii up to 2.7 mm, indicating a DMT-like adhesion regime. This remarkable scaling performance of the R2G adhesive fibril, as shown in Fig. 1c, outperforms the elastomeric fibrils [1] and advanced fibrillar designs such as gradient fibrils [13], mushroom fibrils [4,17,23] and gradient-mushroom fibrils [49]. These other designs experience a significant loss of adhesion strength when scaled up to the millimeter size (Fig. 1c and Fig. S1). Our experimental measurements represent the first systematic validation of the size effect of adhesive fibrils, governed by the ideal cohesive strength, across a wide range of fibril radii from micro to mm scale [1].

The experimental measurements in Fig. 3d also reveal that the adhesion strength of E44-SMP fibrils in the rubber-phase is lower than 100 kPa, significantly below its theoretical strength (420.2 kPa), indicating a JKR-like adhesion regime for the range of fibril radii. Figure 3e depicts the detachment process of an E44-SMP fibril of 1 mm radius under R2G and rubber-phase conditions (Movie S1). Under the R2G condition, the fibril detaches uniformly (Fig. 3e–i), in agreement with the FEA simulation shown in Fig. 3b–i. In the rubber-phase, the fibril detaches gradually from the periphery (Fig. 3e–ii), in agreement with the FEA simulation shown in Fig. 3b–ii. The adhesion of E44-SMP fibrils with radii within the ‘work window’ can be switched, on-demand, by heating and cooling, between the (strong) DMT-like and (weak) JKR-like regimes. The switchability can exceed 20 by harnessing the adhesion regime switching through R2G transition (Fig. 3f).

To assess the repeatability of the R2G adhesive fibrils, repeated tests were conducted (Fig. 3g). Over 30 testing cycles, the adhesion strength varied within $\pm $4% of the mean value in the DMT-like regime, and within about $\pm 2$% in the JKR-like regime, demonstrating excellent repeatability of the R2G adhesive fibrils.

Pull-off tests on SMP adhesive fibrils with various aspect ratios were further conducted. Figure 3h shows that the R2G adhesion strength is independent of the aspect ratios, whereas the rubber-phase adhesion strength decreases slightly as the aspect ratio increases, resulting in a slight increase in adhesion switchability (blue squares in Fig. 3i). However, when compressed to bulging/buckling shapes in the rubber-phase, the adhesion strength of the fibrils is highly sensitive to the aspect ratio when it is below 4, resulting in a rapid increase of the adhesion switchability to the order of 2000 as the aspect ratio increases (red circles in Fig. 3i, see details in Fig. S8). The mechanics of the adhesion of buckled fibrils have been extensively discussed in the literature [1,24]. Here, it was further found that, when the aspect ratio is above 4, the switchability increases slowly with increasing aspect ratio. To balance the requirements for fibril stability, heating time, and sufficient switchability in practical applications, an aspect ratio of 4–5 is chosen for the following studies.

### Characterization of R2G fibril arrays

**Figure 4.**
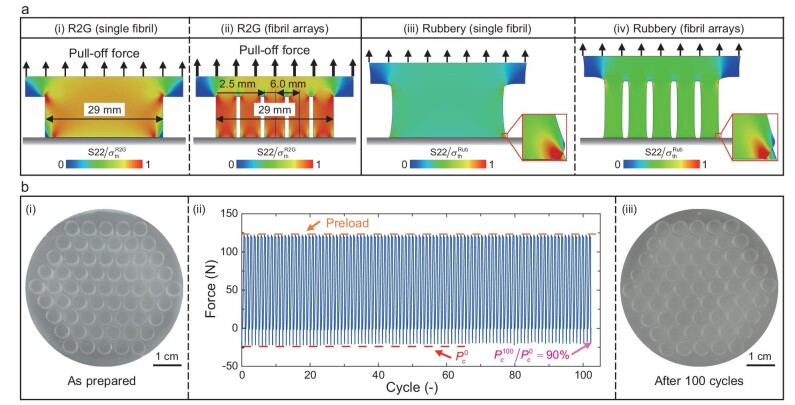
FEA simulation and characterization of the E44-SMP R2G fibrillar adhesive at the array level. (a) Comparison of the 2D-FEA results of the normal stress distributions at the contact interface with comparable apparent contact areas in the (i, iii) single fibril adhesive (*R* = 29 mm) and (ii, iv) fibril arrays (*R* = 2.5 mm, center distance between fibrils *w* = 6 mm) under the R2G conditions (i, ii) and in the rubber-phase (iii, iv). (b) Stability and durability test of the SMP fibril arrays (fibril radius *R* = 2.5 mm and fibril height *h* = 10 mm): (i) photo of the fibril arrays as prepared; (ii) force-cycle curves of the contact-detach test of fibril arrays in rubber-phase; (iii) photo of the fibril array after 100 test cycles.

When the fibril radius exceeds the DMT-limit ${\boldsymbol{R}}_{{\mathrm{cr}}}^{{\mathrm{R}}2{\mathrm{G}}}$, the adhesion regime becomes JKR-like (Fig. 1c), with decreasing strength as the fibril radius increases. One strategy to maintain the load capability (Fig. 1e) and switchability (Fig. 1f) is to use fibril arrays with each fibril below the DMT-limit in radius (Fig. 1b–ii). To understand the mechanisms governing the fibril dimensions in the array, we compared the 2D FEA simulation results of normal stress distribution at the pull-off point of a single fibril $( {R = 29{\mathrm{\ mm}}} )$ and fibril array $( {R = 2.5{\mathrm{\ mm}}} )$ with the same apparent contact area (Fig. 4a).

Figure 4a–i shows that, under R2G conditions, the normal stress is concentrated at the edge of the single fibril adhesive. When the large single fibril is divided into smaller fibrils of the radius within the ‘work window’ (Fig. 3a), the normal stress across the contact interface is more uniform (Fig. 4a–ii), resulting in increased R2G load capability (Fig. 1d). Figure 4a–ii shows that the fibrils, upon loading, detach simultaneously and share the loading equally among themselves (also see experimental validations in Movie S2), resulting in a slow decay of R2G adhesion strength with increasing apparent contact area (Fig. 1e).

On the other hand, when heated to the rubber-phase, Fig. 4a–iii and a–iv show that the normal stress is concentrated at the edge for both the large single fibril and the fibril array, resulting in low adhesion strength and switchability values of ∼20 (Fig. 1f). Controlled buckling of the fibrils further increases switchability by two orders of magnitude (>1000). Moreover, in the rubber-phase, the SMP is soft, leading to large deformation in both the backing and fibrils (Fig. 4a–iv), and failure by progressive detachment of the fibrils from the periphery inwards (also see experimental validations in Movie S2). As a result, the rubbery adhesion strength weakens rapidly with increasing apparent contact area (Fig. 1e).

Bundling of adhesive fibrils during operation and adhesion repeatability affect the performance of these adhesive devices [7]. To evaluate the mechanical stability and adhesion durability of E44-SMP fibrillar adhesives, we carried out repeated contact and detachment tests of E44-SMP fibril arrays (Fig. 4b–i, $R = 2.5{\mathrm{\ mm}},{\mathrm{\ }}h/R = 4$) in the rubber-phase (at 90°C). The adhesion force exhibits good repeatability and durability with only slight degradation (<10%, Fig. 4b–ii) and no bundling, fracture, or wear in the fibrils after 100 cycles (Fig. 4b–iii). Even with aspect ratios of 10 and 20 (Section S10), the SMP fibril arrays demonstrated good stability and repeatability when compressed to buckling during repeated tests.

### Applications of SMP R2G fibrillar adhesives

#### Soft gripper

**Figure 5.**
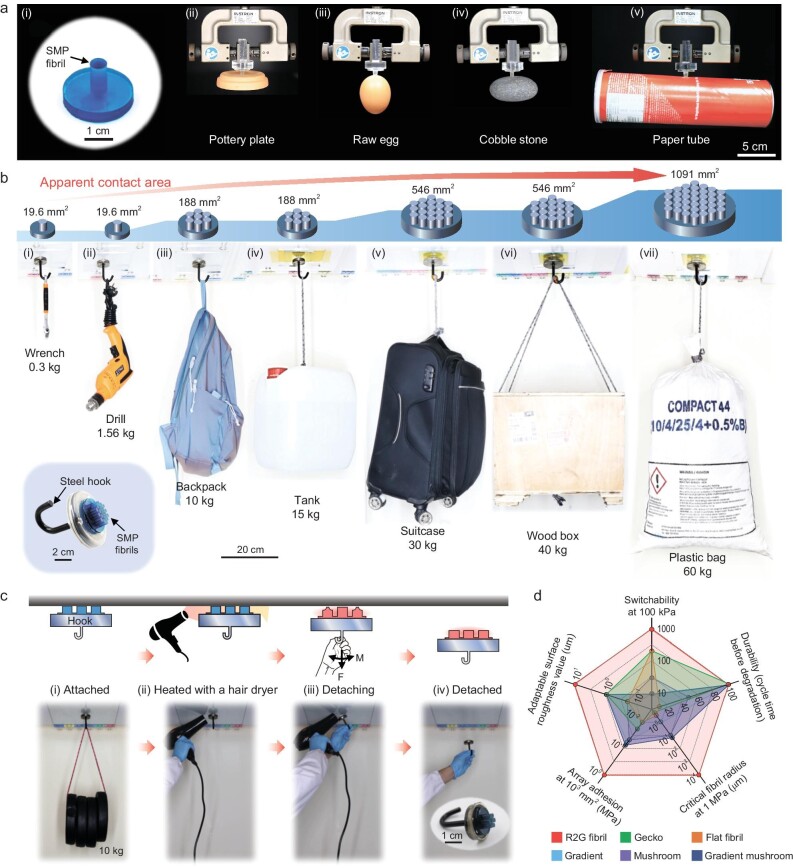
Demonstrations of utilizing the SMP fibrillar adhesives as soft grippers and detachable superglues. (a) Demonstrations of the potential to use (i) the R2G SMP adhesive fibril (*R* = 2.5 mm, *h* = 10 mm) as a soft gripper to grip various objects including (ii) a pottery disk (41 g), (iii) a raw egg (58 g), (iv) a cobble stone (241 g) and (v) a paper tube for potato chips (170 g). (b) Photos of the SMP fibrillar adhesives of various sizes adhered to a glass ceiling and holding heavy objects. (i–ii) A single adhesive fibril can support (i) a wrench (0.3 kg) and (ii) a drill (1.56 kg). (iii–iv) An array of seven fibrils can support (iii) a backpack (10 kg) and (iv) a tank of water (15 kg). (v–vi) An array of 19 fibrils can support (v) a suitcase (30 kg) and (vi) a wood box (40 kg). (vii) An array of 37 fibrils can support a bag of steel weights (60 kg). Inset shows the photo of an SMP fibrillar adhesive hook, the SMP is dyed blue for better visualization. (c) Illustrations and snapshots showing the on-demand detachment process of the SMP fibrillar adhesive hook by heating the SMP fibrils using a hairdryer. (d) Summary and comparison of the performance of different fibrillar adhesive designs. See Section S11 for details.

We demonstrate a soft gripper based on a R2G smart adhesive device. A millimeter-sized adhesive fibril ($R = 2.5{\mathrm{\ mm}},{\mathrm{\ }}h/R = 4$, Fig. 5a–i) is fabricated via molding [26,29], and used for the gripping, moving and placing of various objects (Movie S3). This demonstration showcases the adhesive fibril's strong adhesion and on-demand switchability. With R2G adhesion, a single SMP adhesive fibril can hold glass bottles of 100–1000 ml filled with water, with weights up to 1.66 kg (Fig. S12b). Especially, the SMP adhesive exhibits excellent adaptability to surface roughness [29], allowing the firm holding of objects with diverse surface textures (Fig. 5a). While holding objects in the R2G conditions, the SMP is in the stiff glassy state, ensuring more reliable holding of heavy objects for extended periods of time compared to conventional soft elastomers such as PDMS.

#### Reusable superglue devices

To demonstrate the capabilities of the SMP fibrillar adhesives, we fabricated SMP fibril arrays ($R = 2.5{\mathrm{\ mm}},{\mathrm{\ }}h/R = 4$) arranged in a hexagonal structure (Section S9), and integrated them on a steel plate with a hook (Fig. 5b and Movies S4–S7). Figure 5b demonstrates the load capacities of the devices with different fibril numbers. The smallest one with a single fibril of 19.6 mm^2^ cross-sectional area can support loads of 0.3–1.56 kg (Fig. 5b–i, ii). An array of 19 fibrils can support a box of 40 kg (Fig. 5b–vi and Movie S4). The largest one with 37 fibrils and effective contact area of 1091 mm^2^ can support a weight of 60 kg (∼2307 times the weight of the SMP adhesive, Fig. 5b–vii, Movie S5).

Moreover, the SMP fibril arrays can be quickly heated and detached easily, as depicted in Fig. 5c and Movie S6. When heated with a hairdryer, the time required for the temperature of the fibril arrays to reach 60°C was less than one minute (Figs S13 and S14). Consequently, the SMP fibril array adhesive device can be easily detached upon heating with a hairdryer after use, as demonstrated in Fig. 5c and Movie S7.

### Comparative discussions over gecko and gecko-inspired fibrillar adhesives

Figure 5d provides a comparison of performance of the R2G smart fibrillar adhesives with gecko and gecko-inspired fibrillar adhesives of different designs, such as mushroom shape and modulus gradient fibrils (Section S13). The R2G fibrillar adhesive outperforms the other designs in various aspects, including fibril size, array adhesion strength, surface adaptability, adhesion switchability and durability.

In nature, it is known that the heavier an animal is, the denser and smaller its adhesive pad fibrils [1]. However, increasing fibril density and reducing fibril radius can result in fibril collapse and bundling [1], which ultimately limits the packing density, thus the adhesion strength. Animals that rely on ubiquitous adhesion for locomotion including crawling on ceilings are limited to a maximum body weight of 150 g [1]. Similarly, bio-inspired fibrillar adhesives have faced limitations due to adhesion scaling limits at the fibril/array level [1]. However, the R2G fibrillar adhesive surpasses these limitations and offers several advantages. It provides strong adhesion (>1 MPa) and tunable switchability (>1000) by extending the single fibril DMT regime to millimeter-size, achieving improved adhesion efficiency (>57.8%) of fibril arrays with effective contact area up to 1000 mm^2^. In this way, the R2G fibrillar adhesive surpasses the capabilities of both natural adhesive systems (e.g. gecko feet) as well as their bio-inspired counterparts.

In contrast to the hierarchical fibrils of micro/nano-scale in nature [1], the R2G fibrillar adhesive system presents several advantages with its one-level, millimeter-sized fibrils. First, the fabrication of one-level fibrils of millimeter-scale is relatively straightforward using, e.g. 3D printing or molding methods. Second, while natural systems are limited to an adhesion strength of 100 kPa, the R2G adhesive fibrils can reach an adhesion strength of ∼2 MPa, enabling them to support loads up to tens of kilograms with a small adhesive area. Third, the R2G fibrillar adhesive system can easily avoid lateral collapse and bundling of fibril arrays, resulting in enhanced mechanical robustness and stability.

With these advantages, the R2G fibrillar adhesive systems are expected to find many applications in areas that require supporting heavy loads using adhesives, such as detachable and sustainable adhesive joints [1], reusable superglues [34,40] and robotic grippers [1] for heavy objects, and wall climbing robotics [6,15]. The current R2G method is especially viable for applications that do not require very short attachment and detachment times, such as reusable superglues or detachable joints. Since the current systems using thermally-controlled SMP is limited in actuation speed, applications that require fast response may benefit from incorporating materials with lower transition temperature or other actuation mechanisms [37,46,50] to achieve more rapid switching. Additionally, careful material selection might allow this adhesion paradigm to be extended to extreme environments like hot weather conditions.

## CONCLUSION

In conclusion, this study has introduced a new design paradigm of fibrillar adhesives that have the potential to revolutionize the field of adhesion technology. Overcoming the long-standing trade-off between adhesion strength and adhesion switchability, as well as addressing size scale limitations, these adhesive fibrils based on the R2G transition in SMPs have demonstrated outstanding performance. The adhesion strength achieved, ∼2 MPa, is unprecedented and sets a new benchmark for fibrillar adhesives. Additionally, the switchability of ∼2000 and scalability to the millimeter-scale at the single fibril level mark a significant leap forward in this field. The collective performance of SMP fibrillar adhesive arrays, with switchability exceeding 1000 and an impressive adhesion efficiency of 57.8%, offers a compelling advantage over existing fibrillar adhesives. Their scalability to an apparent contact area of up to 1000 mm^2^ is a testament to their versatility. Beyond laboratory tests, these SMP fibrillar adhesives demonstrate practical utility as soft grippers and reusable superglue devices. Their ability to securely hold and release objects of >2000 times their own weight highlights their potential for a wide range of applications, particularly in high-payload scenarios. In summary, the innovative characteristics of these SMP fibrillar adhesives represent a substantial advancement in the realm of smart fibrillar adhesives. This groundbreaking adhesion design paradigm can extend beyond SMPs, offering avenues for the development of next-generation adhesives employing various phase-change materials. Their exceptional performance opens up possibilities for transformative applications across various industries, especially for high-payload applications, and positions them as a promising solution to longstanding challenges in adhesion technology.

## MATERIALS AND METHODS

### Synthesis of E44-SMP

The E44-SMP was prepared by mixing the liquid crosslinker Poly(propyleneglycol)bis(2-aminopropylether) (Shanghai Aladdin Bio-Chem Technology Co., LTD.) into the liquid E44 monomer (Feicheng Deyuan Chemical Co.) at a mass ratio of 81:46. The mixture was degassed in a vacuum chamber for 30 minutes and then poured into molds and precured in an oven for one hour at 100°C, followed by post-curing for another hour at 130°C. See details in Section S5.

### Modulus characterization of E44-SMP

Dynamic Mechanical Analysis was conducted in film tension mode to measure the storage modulus of the E44-SMP at different temperatures. The Young's modulus values of the SMP in the glass-phase (30°C) and rubber -phase (90°C) were measured using a tensile machine (Instron 5566) with a furnace, under a quasi-static loading of 10 mm/min. See details in Section S5.

### Adhesive parameters measurements of E44-SMP

Pull-off tests were conducted using a flat punch (*R* = 10 mm) on E44-SMP samples (diameter of 100 mm) with various thicknesses to measure the interfacial theoretical strength ${{{\boldsymbol{\sigma }}}_{{\mathrm{th}}}}$ and the work of adhesion ${{{\boldsymbol{w}}}_{{\mathrm{ad}}}}$ of the E44-SMP material under the R2G condition and the rubber-phase. The values of ${{{\boldsymbol{\sigma }}}_{{\mathrm{th}}}}$ and ${{{\boldsymbol{w}}}_{{\mathrm{ad}}}}$ were obtained through non-linear fitting of the theoretical expression for the pull-off force as a function of sample thickness. See details in Section S6.

### FEA simulations

FEA simulations were conducted in ABAQUS/Explicit to capture the adhesion regimes under different fibril radii. An axisymmetric model was used with the fibril height *h* equal to 4 times the fibril radius *R*. Cohesive elements were used to simulate adhesive behavior. A pre-crack with a length of 1/200 of the fibril radius was introduced at the outer edge by deleting one cohesive element. A linear traction-separation law was used to model adhesive interactions. For the FEA simulations of the fibril arrays, 2D models were used with the same modeling parameters. See details in Section S7.

### Adhesion tests

Pull-off tests were conducted to measure the adhesion strength of E44-SMP adhesive fibrils and fibril arrays. During the tests, the E44-SMP fibrillar adhesive was heated to the rubber-phase (90°C) and brought into contact with the glass substrate at a speed of 10 µm/s until a certain preload was reached (the preload was increased until the pull-off force reached a plateau), dwelled for 5 minutes and pulled away at 90°C for rubbery adhesion measurement and at 30°C for R2G adhesion measurement. See details in Sections S8 and S9.

### Stability and repeatability tests

The mechanical stability and adhesion repeatability of the E44-SMP fibril arrays were tested in the rubber-phase with both macro-sized (*R* = 2.5 mm, *h/R* = 5) and micro-sized (*R* = 250 µm, *h/R* = 10, 20, 30) samples. The time-force curve was recorded during the test and optical images were taken before and after the test to check for lateral collapse, sticking, wear and tear. See details in Section S10.

## Supplementary Material

nwae106_Supplemental_Files
