# Supplementary material for: Fibrillar adhesives with unprecedented adhesion strength, switchability and scalability
Source: Natl Sci Rev. 2024 Mar 20;11(10):nwae106. doi: 10.1093/nsr/nwae106 (PMC11413535; doi:10.1093/nsr/nwae106)
Supplement: nwae106_Supplemental_Files [file nwae106_supplemental_files.zip › Supplementary data.pdf]

Supplementary Materials for

Fibrillar adhesives with unprecedented adhesion strength, switchability and scalability

Changhong LINGHU [1], Yangchengyi Liu [1,2], Xudong Yang [1], Dong Li [1], Yee Yuan Tan [1], Haziq Bin Mohamed Hafiz Mohamed [1], Fadhli Bin Rohani Mohammad [1], Zihao Du [1,3], Jiangtao Su [4], Yan Li [1], Yucheng Huo [1], Hanyan Xu [4], Xiufeng Wang [2], Yifan Wang [1], Jing Yu [4],
Huajian Gao [1,5,6*], K. Jimmy Hsia [1,7,#].

[1] School of Mechanical and Aerospace Engineering, Nanyang Technological University, 50 Nanyang Avenue, Singapore 639798, Singapore
[2] School of Materials Science and Engineering, Xiangtan University, Xiangtan, Hunan 411105, China
[3] Department of Engineering Mechanics, Zhejiang University, Hangzhou 310027, China
[4] School of Materials Science and Engineering, Nanyang Technological University, Singapore 639798, Singapore
[5] Institute of High-Performance Computing, A*STAR, Singapore 138632, Singapore
[6] Mechano-X Institute, Applied Mechanics Laboratory, Department of Engineering Mechanics, Tsinghua University, Beijing 100084, China.
[7] School of Chemistry, Chemical Engineering and Biotechnology, Nanyang Technological University, 50 Nanyang Avenue, Singapore 639798, Singapore

[*#] *To whom correspondence should be addressed.*
[*] *gao.huajian@tsinghua.edu.cn;* [#] *kjhsia@ntu.edu.sg*

This file includes:

Supplementary sections

Section S1. Scaling performance of the fibrillar adhesives at the fibril level 2
Section S2. Adhesion switchability versus strength of elastomeric adhesive fibrils 4
Section S3. Scaling performance of fibrillar adhesives at the array level 5
Section S4. Statistics of the elastic modulus and adhesive parameters of typical adhesive materials 6
Section S5. Sample preparation and modulus characterization of E44-SMP 8
Section S6. Pull off tests to measure the adhesive parameters of E44-SMP 9
Section S7. FEA simulations of the adhesion regimes of the SMP adhesive fibril 11
Section S8. Adhesion test of an individual SMP adhesive fibril 13
Section S9. Comparison of the single fibril and fibril arrays 15
Section S10. Mechanical stability and adhesion repeatability of the SMP fibrillar adhesive 18
Section S11. R2G adhesive fibrils as soft grippers 20
Section S12. Monitoring of the heating process of the SMP fibrillar adhesives 21
Section S13. Summary and comparison of the performance of different fibrillar adhesive designs. 23

Figure S1 to S14
Table S1 to S3
Captions for Supplementary Movies S1 to S7

Other Supplementary Material for this manuscript includes the following:

Movies S1 to S7 (.mp4 format)

Section S1. Scaling performance of the fibrillar adhesives at the fibril level

Natural adhesive fibrils are made of stiff materials such as keratin or resilin, with elastic modulus in the range of 1-10 GPa [1-3]. They approach the DMT-like regime mainly by reducing fibril size to the micro/nano-scale [4, 5] and optimizing the tip geometry, such as using a mushroom shape [6, 7]. However, existing synthetic adhesive fibrils are usually made of some soft to moderately stiff materials, such as Polydimethylsiloxane (PDMS) [8-20], Polyurethane (PU) [21-25] and Polyurethane acrylate (PUA) [26-28], with relatively large work of adhesion values (~ 0.1 – 10 J/m^2) in consideration of limitations such as the adaptability to surface roughness and ease of fabrication. The DMT-like regime is usually inaccessible for these materials unless the fibril tip is optimized (e.g., using a mushroom-shaped tip [29-32]) at a moderately small size (e.g., < 10 μm). The maximum adhesion strength of the adhesive fibrils reported so far is summarized in Fig. S1. The adhesion strength decreases with increasing fibril radius, due to stress concentration at the contact interface in the JKR-like adhesion regime. The fibril adhesion strength of homogenous flat fibrils [9, 10, 15, 17, 25, 27, 33-44] scales according to a power law

$$\sigma \propto R^{-0.5089} \text{ ------ (S1)},$$

as the fibril radius increases. The fibril adhesion strength due to Van der Waals interactions changes from about 100 kPa to less than 25 kPa when the fibril radius increases from 100 μm to 1 mm. In nature, the adhesive fibrils are usually not homogeneous but graded with a stiffer steam and a softer tip [45, 46], which has inspired the development of gradient adhesive fibrils [25, 27, 43, 44] that can alleviate stress concentration to enhance adhesion strength (~75 kPa when the fibril radius is scaled to millimeter size**)**. Further optimization of the shape of the fibril tip by using the mushroom shape may allow for a more uniform distribution of stress at the contact interface [8, 9, 11-14, 16, 18-20, 24, 47-49], hence increasing the adhesion strength to around 100 kPa when the fibril radius is scaled to millimeter size. The combination of gradient-modulus and mushroom-shape designs [16, 24, 28, 49] can further increase the adhesion strength of adhesive fibrils to around 200 kPa when the fibril radius is scaled to millimeter size. In spite of these efforts, while scaled to the millimeter scale, current designs still fail to reach an adhesion strength on the order of 1 MPa, which is desired for many high payload applications.

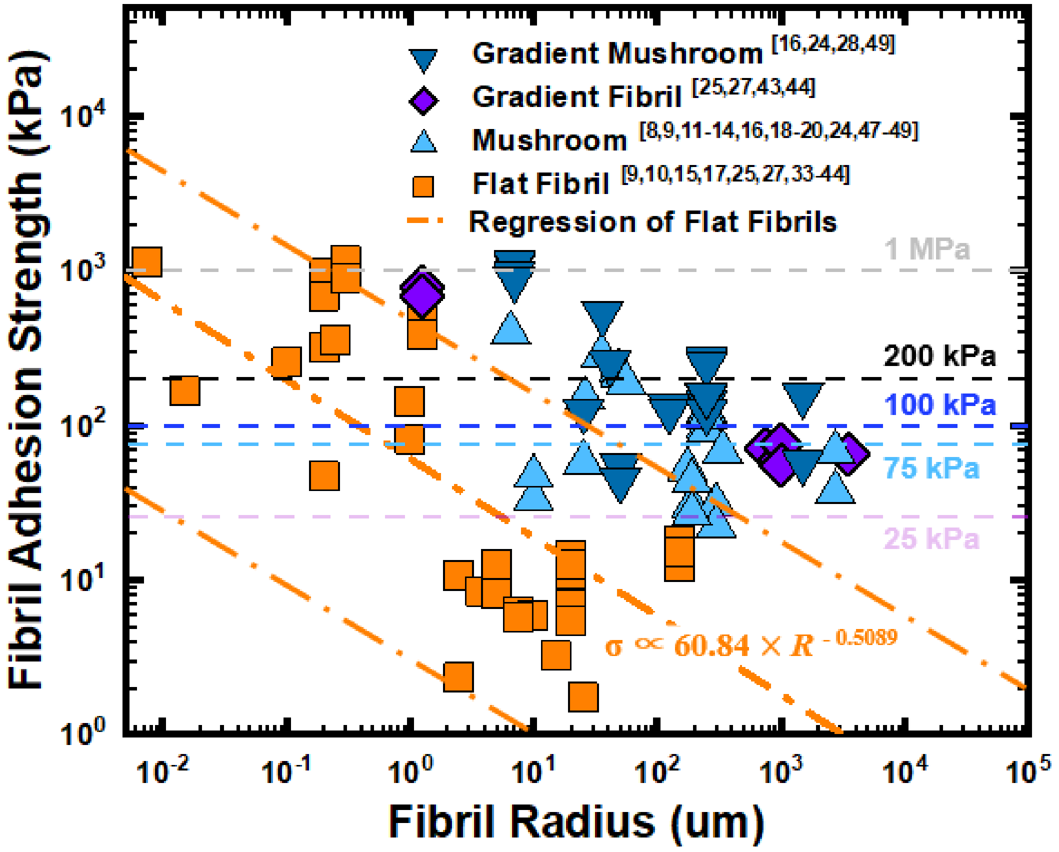

Fig. S1. Statistics of the adhesion strength scaling performance of elastomeric adhesive fibrils under different fibril radii for homogeneous flat fibrils (data collected from refences [9, 10, 15, 17, 25, 27, 33-44]), gradient fibrils (data collected from refences [25, 27, 43, 44]), mushroom fibrils (data collected from refences [8, 9, 11-14, 16, 18-20, 24, 47-49]) and gradient-mushroom (G-M) fibrils (data collected from refences [16, 24, 28, 49]).

Section S2. Adhesion switchability versus strength of elastomeric adhesive fibrils

Existing engineering approaches to realizing switchable adhesion using elastomeric fibrils are generally based on principles such as controlled shearing [18], buckling [23], and peeling [11, 50], etc. One of the major challenges for smart adhesives is the trade-off between adhesion strength and adhesion switchability. The adhesion switchability versus adhesion strength of elastomeric adhesive fibrils are summarized in Fig. S2, according to the actuation methods used to reduce adhesion strength for on-demand detachment. It shows that the adhesion switchability (strength) generally increases at the cost of reduced adhesion strength (switchability). When the adhesion strength is increased to around 2 MPa, the elastomeric adhesives become almost non-switchable.

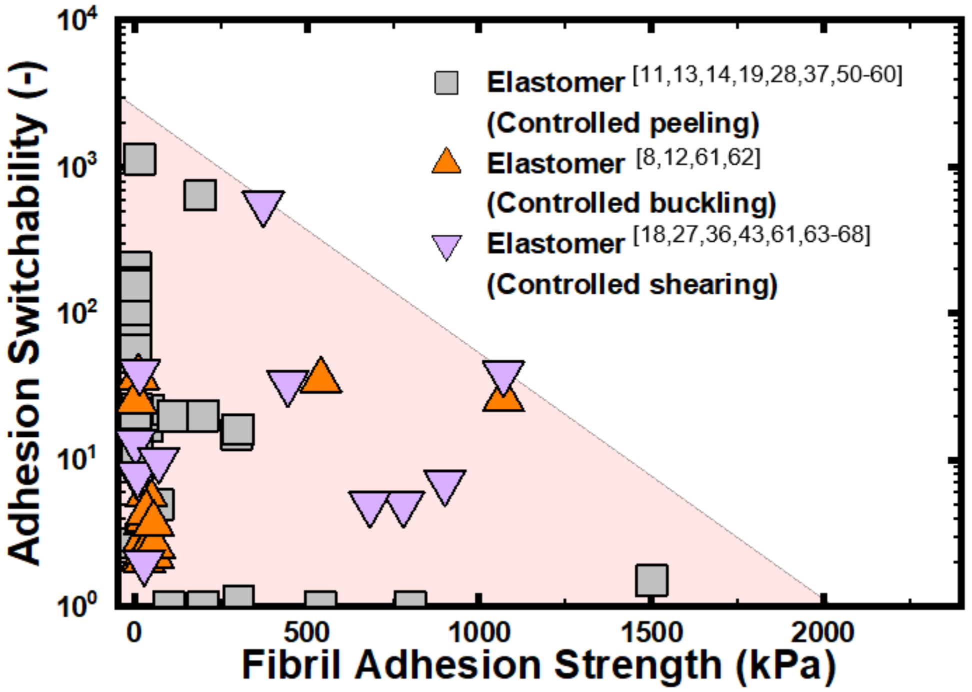

Fig. S2. Summary of the adhesion switchability versus adhesion strength of adhesive fibrils made of elastomers under different adhesion actuation methods. Gray squares - elastomeric adhesive fibrils actuated by controlled peeling [11, 13, 14, 19, 28, 37, 50-60]. Orange upper triangles - elastomeric adhesive fibrils actuated by controlled buckling [8, 12, 61, 62]. Purple lower triangles - elastomeric adhesive fibrils actuated by controlled shearing [18, 27, 36, 43, 61, 63-68].

Section S3. Scaling performance of fibrillar adhesives at the array level

At the array level, the effective adhesion strength $\boldsymbol{\sigma}_{\mathrm{eff}}$ (the adhesion force over the apparent contact area) does not necessarily scale up with the number (or apparent contact area) of fibrils due to possible uneven load sharing among different adhesive fibrils, which presents an additional challenge in the application of fibrillar adhesives. When scaled up at the array level, the effective adhesion strength $\boldsymbol{\sigma}_{\mathrm{eff}}$ of the fibril arrays reduces as the apparent contact area $\boldsymbol{A}_{app}$ increases. The scaling performance of the fibril arrays made of typical elastomeric adhesive materials like PDMS [8-10, 12, 20], Carbon nanotubes (CNT) [34, 37, 69], PU [24, 47] and Polyvinyl Siloxane (PS) [70] are summarized in Fig. S3. Linear regressions of these results show that the load capability $\boldsymbol{P}_{\mathrm{c}}$ and effective adhesion strength $\boldsymbol{\sigma}_{\mathrm{eff}}$ of the reported fibrillar adhesive systems scale according to

$$P_{\mathrm{c}} \propto A_{\mathrm{app}}^{-0.797} \text{ ------ (S2)},$$

$$\sigma_{\mathrm{eff}} \propto A_{\mathrm{app}}^{-0.203} \text{------ (S3)}.$$

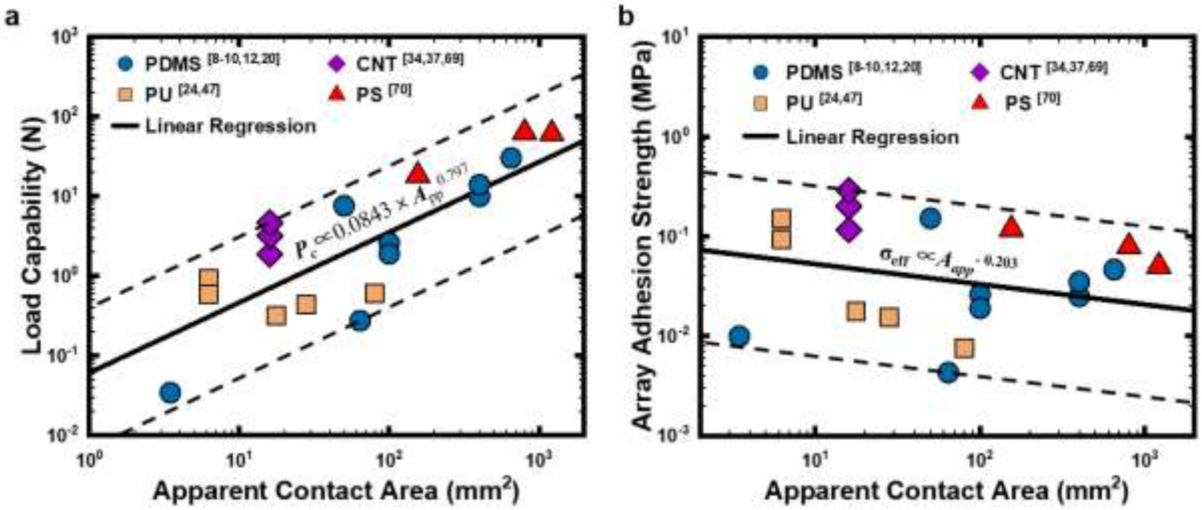

Fig. S3. Summary of **a**, the load capabilities and **b**, effective array adhesion strength (load capability over the apparent contact area) of fibrillar adhesives made of elastomers like PDMS [8-10, 12, 20], CNT [34, 37, 69], PU [24, 47], and PS [70].

Section S4. Statistics of the elastic modulus and adhesive parameters of typical adhesive materials

The reported values of the elastic modulus, adhesion strength and work of adhesion of typical materials used for adhesive fibrils are summarized in Table S1. In nature, adhesive fibrils are made of quite stiff materials such as keratin and resilin. For these materials, the elastic modulus ranges from 1 – 10 GPa [1-3], the reported adhesion strength from 10 – 600 kPa [71-81] and the work of adhesion (on glass) on the order of 10 - 50 mJ/m^2 [82, 83]. Natural adhesive fibrils may approach the DMT-like regime mainly through reduced fibril size [4, 5] and optimized tip shape [6, 7]. One thing worth stressing here is that realizing strong normal adhesion is generally much more challenging than shear adhesion [37]. For example, gecko is known to have a shear adhesion of around 100 kPa [84, 85], but a normal adhesion only of 18 kPa [80]. Synthetic fibrillar adhesives using CNT [34, 37, 69] are quite similar to natural adhesive fibrils, which reach the DMT-like regime with strong adhesion through very small fibrils radius (~ 10 nm) and large elastic modulus (1000 GPa). However, the CNT adhesives show little adhesion switchability. In contrast, most existing synthetic fibrils are made of some soft to moderately stiff materials (~100 kPa – 100 MPa, such as PDMS [8-20], PU [21-25], PUA [26-28, 86]) with relatively large work of adhesion (~ 0.1 – 10 J/m^2) in consideration of other factors such as roughness adaptability and ease of fabrication. The normal adhesion strength values of these synthetic fibrils are usually smaller than 1 MPa. In contrast, materials like hydrogels [87-89] and shape memory polymers (SMPs) [90-96] have a large range of tunable elastic modulus (~ 10 kPa – 10 GPa), normal adhesion strength (~ 10 kPa – 10 MPa) and work of adhesion (~ 1 - 1000 J/m^2). While hydrogels are susceptible to stability issues due to dehydration over time, epoxy SMPs [93, 94, 96] are very stable, exhibit dramatically tunable modulus (~100 kPa – 10 GPa) upon rubber-to-glass (R2G) transition, and achieve very strong adhesion on most materials (~ MPa), making them an ideal candidate as smart adhesive fibrils.

Table S1. Summary of the elastic modulus and adhesive parameters (on glass substrate) for typical adhesive materials

Materials	Elastic Modulus	Adhesion Strength	Work of adhesion
**Animals (Keratin or Resilin)**	1 – 10 GPa<br>Ref. [1-3]	10 – 600 kPa<br>Ref. [71-81]	10 - 50 mJ/m^2<br>Ref. [82, 83]
**CNT**	1000 GPa<br>Ref. [34, 37, 69]	166 kPa – 1.13 MPa<br>Ref. [34, 37, 69]	20 mJ/m^2 – 4.5 J/m^2<br>Ref. [34, 37, 69]
**PDMS**	24 kPa – 12 MPa<br>Ref. [97-100]	1.74 kPa – 397 kPa<br>Ref. [8-15, 18, 19]<br>47 kPa – 955 kPa<br>Modified PDMS [16, 20, 35, 43, 44]	40 mJ/m^2 – 18 J/m^2<br>Ref. [101-105]
**PU**	172 kPa – 9 MPa<br>Ref. [24, 25, 47, 106, 107]	34.7 kPa – 528.7 kPa<br>Ref. [21, 22, 24, 25, 108]	3.2 J/m^2 – 71.9 J/m^2<br>Ref. [24]
**PUA**	19.8 MPa – 110 MPa<br>Ref. [26-28, 86]	260 kPa – 1.11 MPa<br>Ref. [26-28, 86]	24 mJ/m^2 – 3.8 J/m^2<br>Ref. [26, 86]
**Hydrogel**	4.5 kPa – 600 MPa<br>Ref. [70, 109, 110]	5 kPa – 500 kPa<br>Hydrated [87, 89, 111]<br>1.89 MPa – 3.64 MPa<br>Dehydrated [88]	1 J/m^2 – 1 kJ/m^2<br>Ref. [87, 109, 112]
**Epoxy SMP**	80 kPa – 5 GPa<br>Ref. [93, 94, 96]	6 kPa – 12.4 MPa<br>Ref. [90, 92, 95, 96]	80 mJ/m^2 – 18 kJ/m^2<br>Ref. [96, 113, 114]

Section S5. Sample preparation and modulus characterization of E44-SMP

Sample Preparation: To demonstrate the feasibility of our adhesive paradigm, a thermally-controlled epoxy SMP (E44-SMP [93]) was selected as the adhesive material. The E44-SMP was prepared by mixing the liquid crosslinker Poly(propyleneglycol)bis(2-aminopropylether) (from Shanghai Aladdin Bio-Chem Technology Co., LTD in China) into the liquid E44 monomer (from Feicheng Deyuan Chemical Co. in China) at a molecular ratio of 1:1 (mass ratio of approximately 81:46). The mixture was then degassed in a vacuum chamber for 30 minutes. After degassing, the liquid mixture was poured into molds and precured in an oven for one hour at 100 ℃, followed by post-curing for another hour at 130 ℃.

Modulus Characterization: SMP samples with a rectangle shape (30 mm in length, 6 mm in width and 2 mm in thickness) were prepared using moulding methods. Dynamic Mechanical Analysis (DMA) of the SMP materials were conducted to measure the storage modulus *E*' (unless stated otherwise, storage modulus in this report refers to tensile storage modulus) under different temperatures. Fig. 2c shows the storage modulus of the SMP under different temperatures. At the room temperature (25 – 30 ℃), the SMP is in the stiff glass-phase (*E*' ~ 2 GPa). When heated above the glass transition temperature (~ 45 ℃), the SMP shows a sharp decrease in storage modulus. At around 90 ℃, the storage modulus falls below 1 MPa. Tensile tests were further carried out on standard universal tensile test machine (Instron 5566) with a furnace under 30 ℃ and 90 ℃. Fig. 2d shows the typical stress-strain curves, where the SMP shows an elastic-plastic behaviour in the glass-phase (30 ℃). In consideration of the small deformation in the stiff glass-phase, the SMP is regarded as a linear elastic material in the glass-phase. In the rubber-phase, the SMP shows a hyperelastic behaviour which can be described by the first-order Ogden–Roxburgh model [87, 115]:

$$W_{\mathrm{ela}} = W_{\mathrm{dev}} + W_{\mathrm{vol}} = 2\mu/\alpha^2\left(\bar{\lambda}_1^{\alpha} + \bar{\lambda}_2^{\alpha} + \bar{\lambda}_3^{\alpha}\right) + \frac{1}{D}(J-1)^2 \quad \text{------ (S4)},$$

where W_{ela} is the strain energy density, W_{dev} and W_{vol} its deviatoric and volumetric parts, K_0 = 2/D the initial bulk modulus, μ the initial shear modulus, α the Ogden parameter, $\bar{\lambda}_i = J^{-1/3}\lambda_i$, λ_i the ith principal stretch (i = 1, 2, 3) and J the total volume change. The fitted parameters are μ = 0.1785 MPa, α = 2.7265 and D = 0.2256 MPa^{-1}.

Section S6. Pull off tests to measure the adhesive parameters of E44-SMP

Pull off tests using a flat punch (R = 10 mm) on E44-SMP samples (diameter in 100 mm) with various thicknesses were conducted to measure the interfacial theoretical strength $\boldsymbol{\sigma}_{\mathrm{th}}$ and the work of adhesion $\boldsymbol{w}_{\mathrm{ad}}$ of the E44-SMP material under the R2G condition and the rubber-phase. A home-made testing platform was constructed, as illustrated in Fig. S4a. Attached to the Instron 5566, the testing platform was equipped with linear and tip/tilt stages to ensure parallel alignment between the flat punch and the SMP samples. Fig. S4b shows surface morphologies of the glass punch (Fig. S4b-i) and the E44-SMP sample (Fig. S4b-ii), which are both very smooth with an arithmetic mean roughness value of Sa = 1.752 nm and Sa = 1.870 nm, respectively. The test processes are illustrated in Fig. S4c. For the test of the R2G adhesion (Fig. S4c-i), the SMP was heated to the rubber-phase (90 ℃) first. Then the glass punch was attached to the rubber-phase SMP at a constant speed of 10 μm/s until a certain preload (the preload was increased until a pull-off force plateau is reached, as shown in Fig. S4d), dwelled for 5 minutes and cooled down to the glass-phase. Finally, the glass punch was pulled away at a speed of 100 μm/s, with the measured maximum force giving the pull-off force. The adhesion tests of rubbery adhesion (Fig. S4c-ii) were the same as those for the R2G adhesion, except that the SMP is kept in the rubber-phase throughout the process.

The values of $\boldsymbol{\sigma}_{\mathrm{th}}$ and $\boldsymbol{w}_{\mathrm{ad}}$ can be obtained through non-linear fitting of the theoretical expression for the pull-off force P_{c} as a function of sample thickness t, as shown in Fig. 2e [116], according to

$$P_{\mathrm{c}} = \sigma_{th} \cdot \pi R^2 \times \frac{1 - \exp\left(-\sqrt{\dfrac{32 w_{ad} E}{\pi R \sigma_{th}^2 \left(1-v^2\right)}}\,\psi\left(\dfrac{t}{R}, v\right)\right)}{1 + \exp\left(-\sqrt{\dfrac{18 w_{ad} E}{\pi R \sigma_{th}^2 \left(1-v^2\right)}}\,\kappa\left(\dfrac{t}{R}, v\right)\right)} \quad \text{------ (S5)},$$

where t is the thickness of the E44-SMP substrate, R is the punch radius; E and v are the elastic modulus and Poisson's ratio of the E44-SMP substrate, ψ and κ are dimensionless functions of t/R and v. The validity of Eq. (S5) has been established across various regimes, including the JKR-like, DMT-like, and transitional regimes in between[116]. While only one adhesive parameter can be determined in the limit regimes, both values of $\boldsymbol{\sigma}_{\mathrm{th}}$ and $\boldsymbol{w}_{\mathrm{ad}}$ can be obtained by covering the transitional regime in experiments rather than focusing solely on the limit cases. Experimental data can be fitted to Eq. (S5), and the values of $\boldsymbol{\sigma}_{\mathrm{th}}$ and $\boldsymbol{w}_{\mathrm{ad}}$ can be determined by fitting experimental data to Eq. (S5) and are given in Fig. 2f.

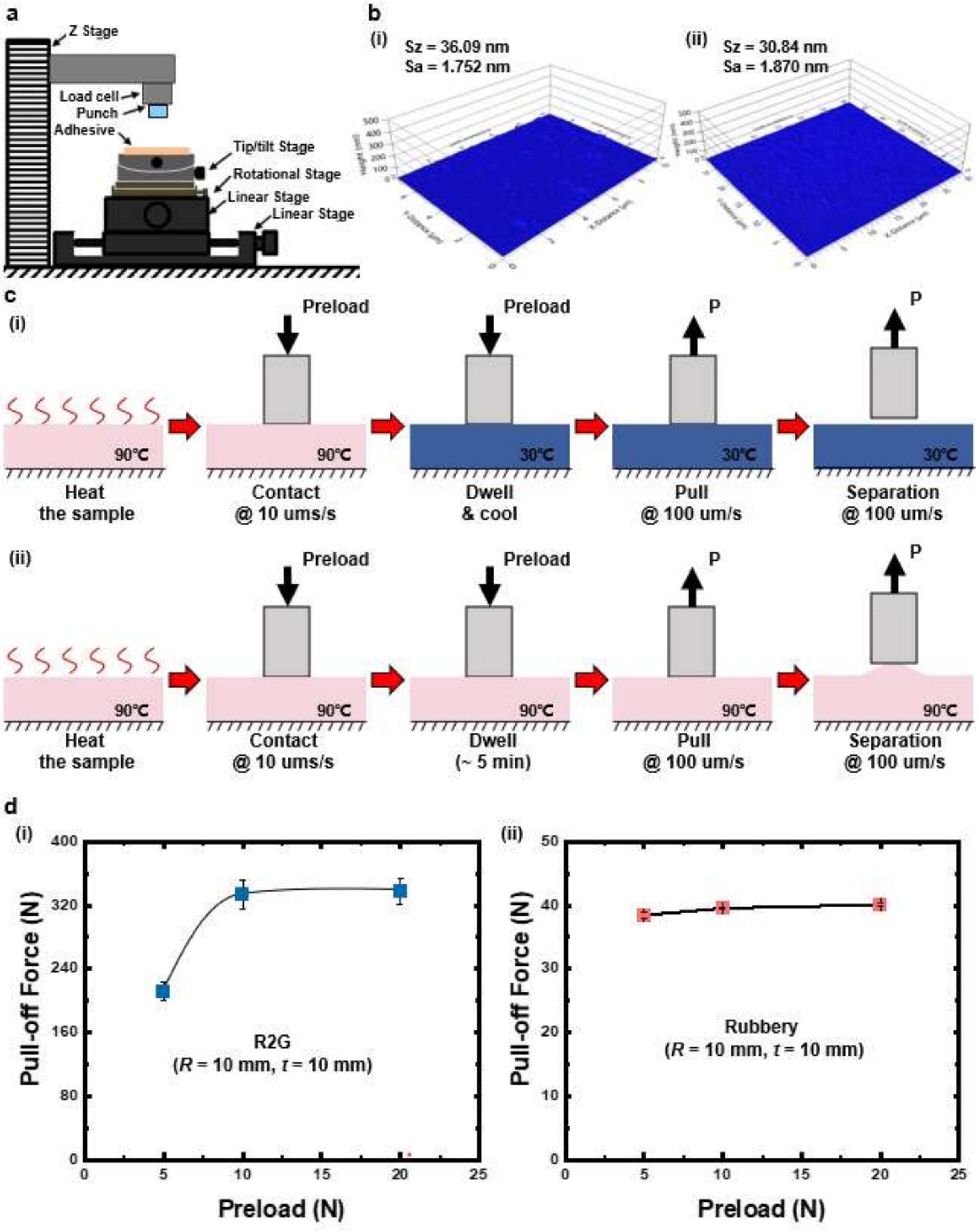

Fig. S4. Details for the measurement of the adhesive parameters of the E44-SMP. **a**, Illustration of the pull-off test platform. **b**, Surface morphology of the (i) glass punch and (ii) the E44-SMP sample. **c**, Illustrations of the measurement processes of the (i) R2G and (ii) rubbery adhesion parameters. **d**, Representative curves showing the dependence of the pull-off force on the preload under the (i) R2G condition and (ii) rubber-phase (*R* =10 mm, *t* = 10 mm).

Section S7. FEA simulations of the adhesion regimes of the SMP adhesive fibril

Finite Element Analysis (FEA) simulations were conducted using ABAQUS/Explicit and a cohesive zone model to capture the adhesion regimes for different fibril radii (as shown in Fig. S5). An axisymmetric model with fibril height h equals to 4 times of the fibril radius R is used, as shown in Fig. S5a. The E44-SMP was regarded as a linear elastic material with an elastic modulus of 2 GPa and Poisson's ratio of 0.35 in the glass-phase, and a hyperelastic material described by the first-order Ogden model in the rubber-phase (μ = 0.1785 MPa, α = 2.7265 D = 0.2256 MPa^{-1}). A thin layer of cohesive elements was used to simulate the adhesive behavior. The cohesive elements were tied to the bottom of the SMP fibril on the top surface and fixed at its bottom surface. A pre-crack with the length of 1/200 of the fibril radius was introduced at the outer edge by deleting one cohesive element. A linear traction-separation law was used to model the adhesive interactions, as shown in Fig. S5b. The stiffness of the cohesive element was set as $K = 6\sigma_{\mathrm{th}}^{\ 2} / w_{\mathrm{ad}}$ to ensure a left triangular, and a maximum nominal stress criterion $Max\,\{\langle t_{\mathrm{n}}\rangle/\sigma_{\mathrm{th}}, t_{\mathrm{s}}/\sigma_{\mathrm{th}}\} = 1$ was used for damage initiation. The SMP was modeled with CAX4R elements, and the minimum element size was set to 1/200 of the fibril radius. The cohesive layer was modeled with COHAX4 elements, and the element length set to the minimum element size of the SMP. Detailed parameters of the FEA simulations are given in Table S2.

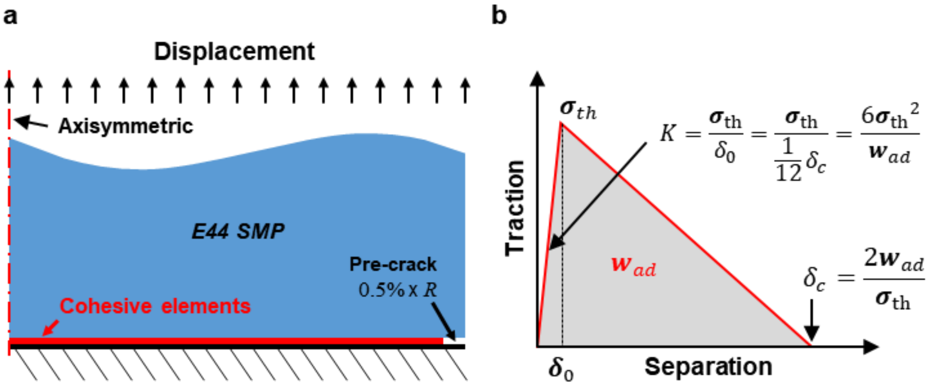

Fig. S5. Detailed setup for the FEA simulations. **a**, Illustration of the FEA simulation setup. **b**, Linear traction-separation law used to model the adhesive interactions.

Table S2. Summary of the modeling parameters for FEA simulations.

State	Elastic behavior	Density	Theoretical adhesion strength	Work of adhesion	K (Stiffness of cohesive elements)
R2G<br>90°C => 30°C	E = 2000 MPa;<br>v = 0.35	2×10^{-9} ton/mm^3	1.893 MPa	1.61×10^{-3} mJ/mm^2	1.335×10^{4} N/mm^3
Rubbery<br>90°C	Ogden Model<br>μ = 0.1785 MPa,<br>α = 2.7265<br>D = 0.2256 MPa^{-1}	2×10^{-9} ton/mm^3	0.4202 MPa	20.27×10^{-3} mJ/mm^2	52.26 N/mm^3

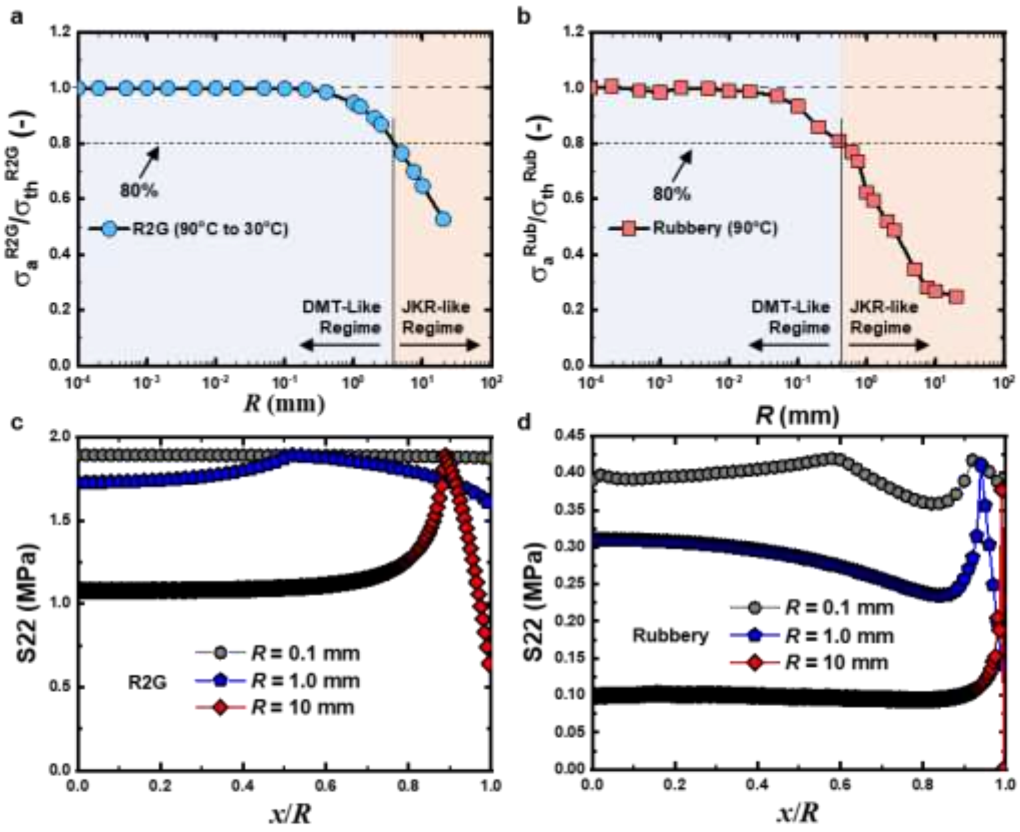

Fig. S6. FEA simulation results of SMP fibrils. **a**, calculated R2G adhesion strength normalized by the R2G theoretical adhesion strength $\boldsymbol{\sigma}_{th}^{R2G} = 1.893\ MPa$ and **b**, calculated rubbery adhesion strength normalized by the rubbery theoretical adhesion strength $\boldsymbol{\sigma}_{th}^{Rub} = 0.4202\ MPa$ under various fibril radii. The adhesion regime is regarded as DMT-like when the adhesion strength is larger than 80% of the theoretical value. **c-d**, Typical normal adhesion stress distribution at the contact interface under **a,** the R2G condition and **b,** rubber-phase.

Figures S6a-b show the FEA results of the fibril adhesion strength under various fibril radii and Figs. S6c-d the normal stress distribution under typical fibril radii. Under the R2G condition, the adhesion regime is DMT-like with an adhesion strength larger than 80% of the theoretical value (Fig. S6a) and a uniform stress distribution when the fibril radius is smaller than 3 mm (e.g., R = 0.1 and 1 mm in Fig. S6c), and JKR-like with stress concentration at the edge part when the fibril radius exceeds 3 mm (e.g., R = 10 mm in Fig. S6c), resulting in rapid decay of adhesion strength with increasing fibril radius (Fig. S6a). In the rubber-phase, the adhesion regime is DMT-like with an adhesion strength larger than 80% of the theoretical value (as shown in Fig. S6b) and a uniform stress distribution when the fibril radius is smaller than 0.4 mm (e.g., R = 0.1 mm in Fig. S6d). When the fibril radius is larger than 0.4 mm, the adhesion regime is JKR-like with stress concentration at the edge part (e.g., R = 1 mm and 10 mm in Fig. S6d), leading to rapid decay of adhesion strength with increasing fibril radius.

Section S8. Adhesion test of an individual SMP adhesive fibril

To further validate the designed 'work window' of our SMP adhesive fibrils, SMP fibrils with different radii R (0.2, 0.5, 1, 2.5, 5, 7.5 and 10 mm) and an aspect ratio of $h/R = 4$ were fabricated through molding [96]. Pull off tests were conducted to measure the adhesion strength of SMP fibrils with different radii. Adhesion measurement details of the SMP adhesive fibrils are shown in Fig. S7. During the pull off tests, the SMP fibril was heated to the rubber-phase (90 ℃) and brought into contact with the glass substrate at a speed of 10 μm/s until a sufficiently large preload, dwelled for 5 minutes and then pulled away under 90 ℃ for rubbery adhesion measurement (Fig. S7a) and under 30 ℃ for R2G adhesion measurement (Fig. S7b). The adhesion strength is given by the pull-off force over the contact area. The preload was increased until the pull-off force reaches a plateau, as shown in Fig. S7c and Fig. S7d.

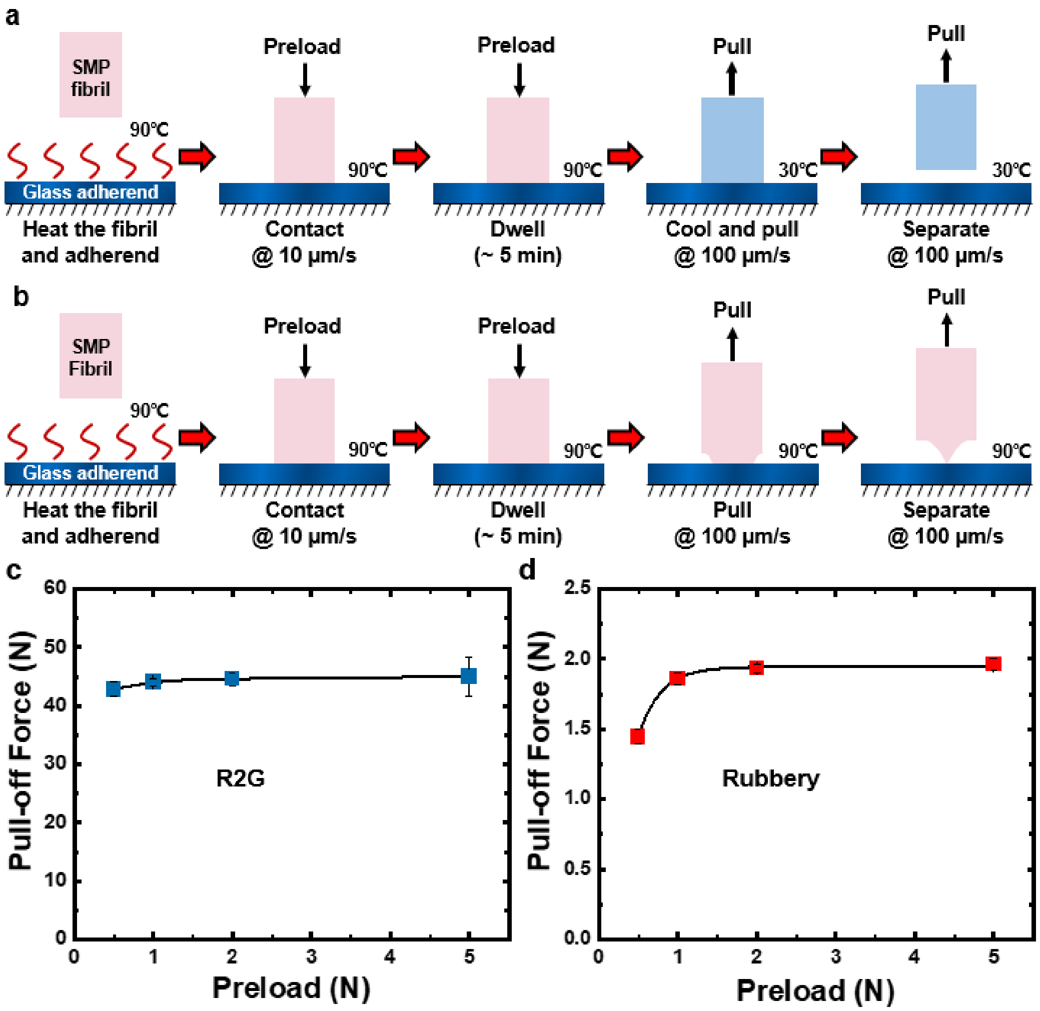

Fig. S7. Details of the adhesion test of a single SMP fibril. **a-b,** Illustrations showing the test process of the adhesion strength under the **a**, R2G condition and **b**, rubber-phase. **c-d**, Representative curves showing the dependence of the R2G and rubbery pull-off forces of a single SMP fibril (R =2.7 mm, h = 10 mm) on the preloads.

In the rubber-phase, the adhesion strength of the single SMP adhesive fibril can be further reduced by a large compression until it bulges or buckles. When the aspect ratio is small than 4 (e.g., 2, see Fig. S8a-i), the fibril bulges under a large compression, as shown in Fig. S8a-ii. Further increase of compression will lead to bulging of the fibril (Fig. S8a-iii) but no compression-induced detachment is observed. The adhesion strength reduces gradually with increasing compression preload and plateaus in the end (Fig. 8a). On the contrary, when the aspect ratio is larger than 4 (e.g., 4, see Fig. S8b-i), the fibril will be compressed to buckling under a large preload. After buckling, the SMP fibril will tilt and detach, leaving a small residual contact between the SMP adhesive fibril tip and the adhered, as shown in Fig. S8b-ii, leading to a sharp drop of the adhesion strength (Fig. 8b).

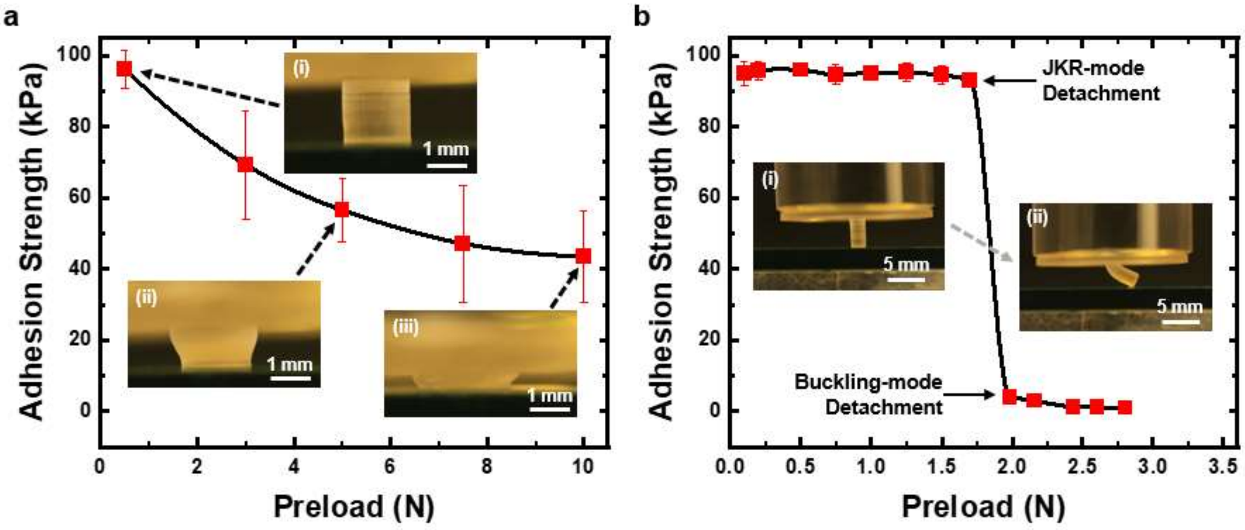

Fig. S8. Measured adhesion strength of an individual SMP fibril under different preloads in the rubber-phase under **a,** a small (R = 1 mm, h = 2 mm) and **b**, large (R = 1 mm, h = 4 mm) aspect ratio. Insets show photographs of the SMP fibril under different preloads.

Section S9. Comparison of the single fibril and fibril arrays

The SMP fibril arrays and their single fibril counterparts (as shown in Fig. S9) with comparable apparent contact areas were prepared utilizing molding as described in the reference [96]. The SMP fibril arrays (Fig. S9a) are characterized by smaller SMP fibrils (R = 2.5 mm, h = 10 mm) arranged in a hexagonal pattern (center to center distance of 6 mm) on an SMP backing of 5 mm thickness. The SMP single fibril adhesives are cylinders fabricated on the larger SMP backing plates of 2 mm thickness. The backing plates are designed to enhance the contact area between the adhesives and the backing glasses for fixation convenience. The total height of the SMP single fibril adhesive including the backing is 15 mm, equal to the total height of the SMP adhesive fibril arrays. For fibril arrays, the contact area $\boldsymbol{A}_{\mathrm{c}}$ is the sum of the areas of every single fibril tip area (bule part as shown in the illustrations in Fig. S9a) and the apparent contact area $\boldsymbol{A}_{\mathrm{app}}$ is the whole area covered by the array (brown part as shown in the illustrations in Fig. S9a). For the SMP single fibril adhesives (see Fig. S9b), the apparent contact area is identical to the contact area, both equal to the tip area of the fibril.

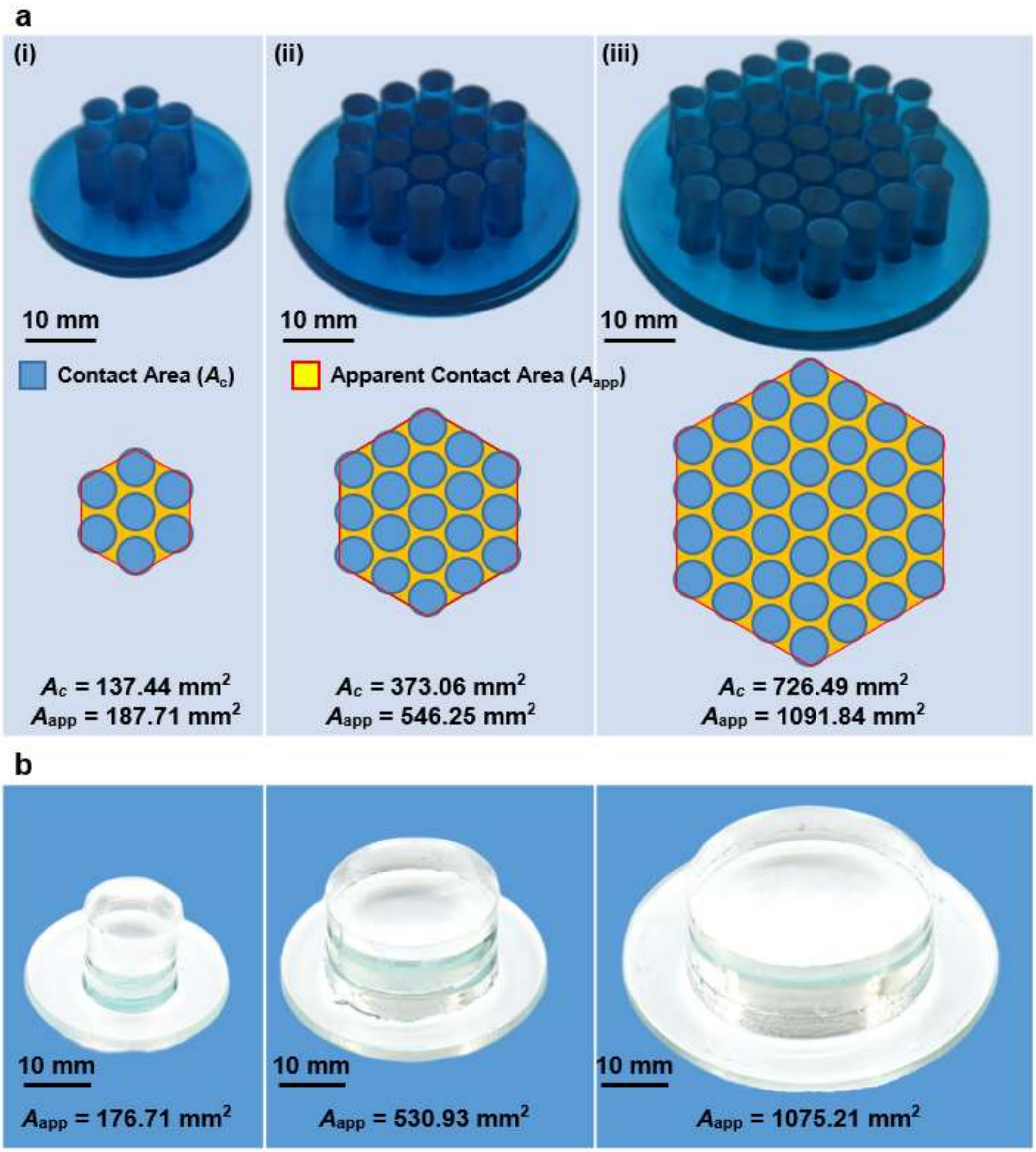

Fig. S9. Photos of **a**, the SMP fibril arrays and **b**, single fibril adhesives with comparable apparent contact areas. The apparent contact area of the SMP fibril arrays is defined by the total area covered by the array.

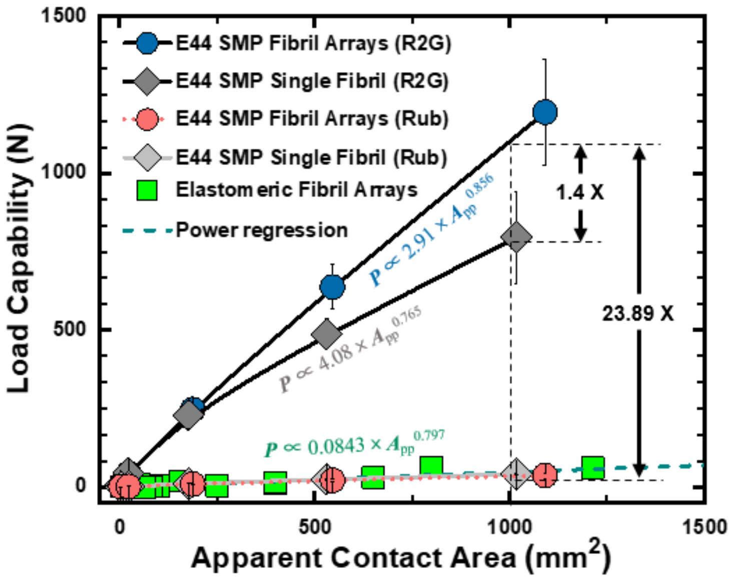

Fig. S10. Scaling performance of the load capabilities of E44-SMP R2G fibril arrays compared to those of E44-SMP single fibrils and existing elastomeric fibril arrays. Data for elastomeric fibrils are from literatures: PDMS [8-10, 12, 20], CNT [34, 37, 69], PU [24, 47], and PS [70].

Figure. S10 shows the measured load capabilities of the SMP R2G fibril arrays, SMP R2G single adhesive fibrils under the R2G condition as compared that in the rubber-phase, with those of the elastomeric fibril arrays as control. As the apparent contact area increases, the R2G pull-off force of the SMP R2G fibril arrays $P_{\mathrm{c-R2G}}^{\mathrm{Fibril\ Arrays}}$ and SMP R2G single adhesive fibril $P_{\mathrm{c-R2G}}^{\mathrm{Single\ Fibril}}$ scales as

$$P_{\mathrm{c-R2G}}^{\mathrm{Fibril\ Arrays}} \propto 2.91 \times A_{app}^{0.856} ------ \text{(S6)},$$

$$P_{\mathrm{c-R2G}}^{\mathrm{Single\ Fibril}} \propto 4.08 \times A_{app}^{0.765} ------ \text{(S7)}.$$

In contrast, the rubbery adhesion forces of both single SMP fibrils and SMP fibril arrays are much smaller than their R2G values and similar to those of the elastomeric fibrillar adhesives $P_{\mathrm{c}}^{\mathrm{Ela}}$ as

$$P_{\mathrm{c}}^{\mathrm{Ela}} \propto 4.08 \times A_{app}^{0.765} ------ \text{(S8)},$$

When the apparent contact area is scaled to 1000 mm^2, the load capability (i.e., pull-off force) of the SMP R2G fibril arrays is 1.4 folds of the SMP R2G single adhesive fibril and 23.89 folds of the elastomeric fibril arrays.

Section S10. Mechanical stability and adhesion repeatability of the SMP fibrillar adhesive

Lateral collapse and bundling problems of adhesive fibrils [33, 85, 117] have been a big challenge to the mechanical stability and adhesion repeatability of fibrillar adhesives since they were first proposed [33] in 2000s. The adhesive fibrils are susceptible to sticking to each other, especially when the fibril radius is small, aspect ratio large and the elastic modulus low. While low modulus can result in very low adhesion in the rubber-phase [118], it is not commonly used in fibrillar adhesives due to this limitation.

It was found that for a given ratio between the fibril center distance *w* and fibril radius *R*, the critical aspect ratio under which lateral collapse does not occur (the so-called static stability) is given by [33, 85, 117]

$$\left(\frac{h}{R}\right)_c < \left(\frac{3^3\pi^4}{2(1-v^2)}\right)^{1/12} \cdot \left(\frac{w}{R}\right)^{1/2} \cdot \left[\frac{E_f R}{\gamma_s}\right]^{1/3} \text{------ (S6)},$$

where h is the height, E_f and v the elastic modulus and Poisson's ratio of the adhesive fibrils, respectively, and γ_s is the work of adhesion (on the order of 300 J/m^2) between adhesive fibrils. According to Eq. (S6), larger fibrils can have bigger aspect ratios, which has the advantage of larger contact compliance to adapt to surface textures and a smaller preload to actuate buckling for detachment. Fig. S11 shows the critical aspect ratio values for static stability of fibril arrays under a given center distance among fibrils as w/R = 4. When the fibril radius is on the order of microns, the fibril aspect ratio should remain below 10 or lateral collapse will occur. In contrast, the critical aspect ratio can exceed 10 with a fibril radius on the order of tens of microns and 30 with a fibril radius on the order of hundreds of microns.

Figure 4c has already shown the mechanical stability and repeatability of the E44-SMP fibril arrays with a millimeter-sized radius (R = 2.5 mm) and a small aspect ratio (h/R =5). To inspect the static and dynamic stability (here dynamic stability refers to the stability after repeated contact and detach tests) of the E44-SMP fibril arrays with large aspect ratio values compressed to buckling, samples (R = 250 μm, h/R = 10, 20, 30) were fabricated and they all showed good static stability as fabricated (Fig. S11-b, i, iii, v). Repeated contact and detachment tests with a large compression displacement of 1 mm (under which all the adhesive fibrils are buckled) were conducted in the soft, rubber-phase at a test temperature of 90 ℃. After 100 test cycles, the adhesive fibrils did not stick to each other and show little observable fracture or wear for samples with aspect ratios of 10 and 20 (Fig. S11b, ii, iv). Under an aspect ratio (h/R = 30) close to the static stability limit, the fibrils stick to each other after 100 cycled tests (Fig. S11b, vi).

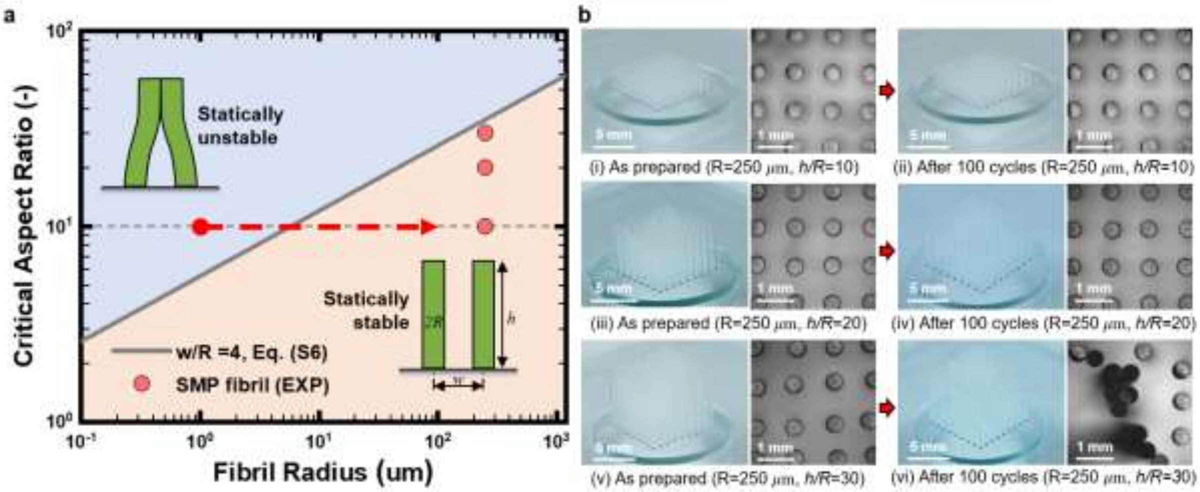

Fig. S11. Static and dynamic stability of the SMP fibril arrays with large aspect ratios. **a**, Theoretical predictions of the critical aspect ratio values under which lateral collapse and bundling of the SMP fibrils in the rubber-phase do not occur under static conditions. **b**, Photos and microscope images showing the SMP fibril arrays as prepared (i – h/R = 10, iii – h/R = 20, v – h/R = 30) and after 100 repeated test cycles (ii – h/R = 10, iv – h/R = 20, vi – h/R = 30). Fibril radius R = 250 μm and compression displacement = 1 mm. all of the fibrils are compressed to buckling in each test.

Section S11. R2G adhesive fibrils as soft grippers

As a preliminary demonstration of the R2G smart adhesive, a millimeter-sized adhesive fibril (*R*=2.5 mm, *h/R*=4) was fabricated and used as a soft gripper for the gripping, moving, and placing of various objects, with a heating gun as the heating source, as shown in Fig. S12. These results prove strong adhesion and on-demand switchability of the R2G adhesive fibril.

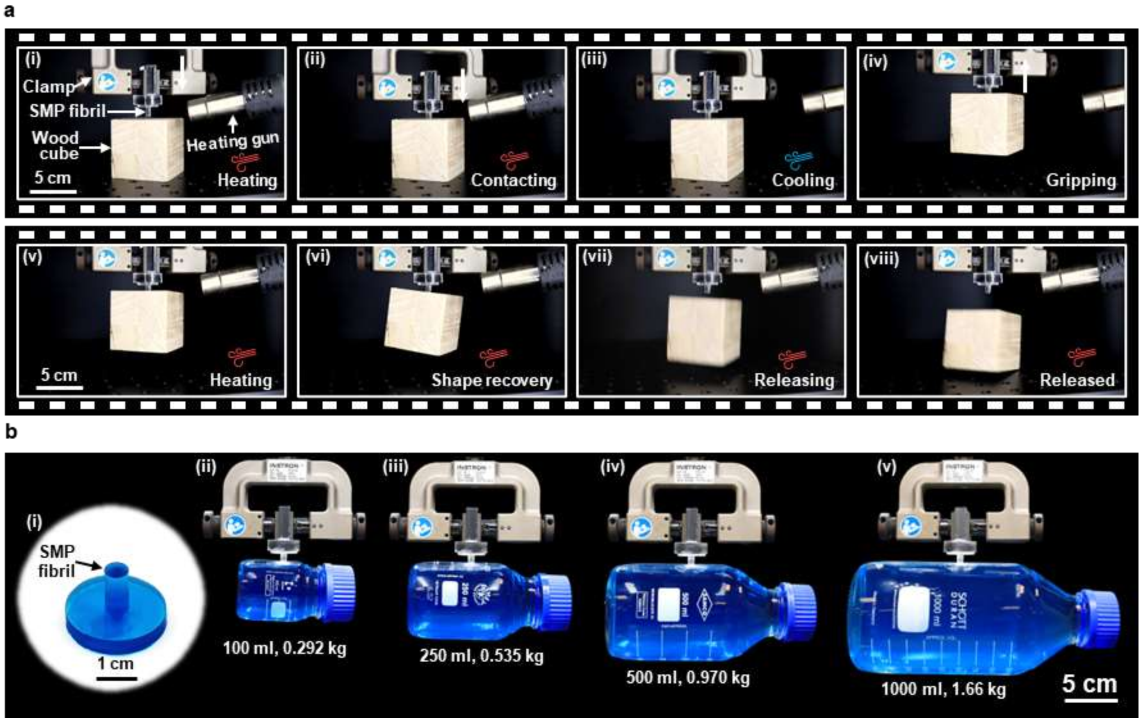

Fig. S12. Demonstrations of the potential to use the R2G SMP adhesive fibril (*R* = 2.5 mm, *h* = 10 mm) as a soft gripper to grip heavy and rough objects. **a,** Gripping a wood cube (60 mm × 60 mm × 60 mm, 64 g) using the SMP adhesive fibril. (i-iv) Gripping process - (i) heating the SMP adhesive fibril, (ii) contacting with the wood cube (preload = 254.6 kPa), (iii) cooling the SMP adhesive fibril to the glass-phase, (vi) gripping the wood cube. (v-viii) Release process - (v) heating the SMP adhesive fibril, (vi) shape recovery of the SMP adhesive fibril, (vii) releasing the wood cube, (vii) wood cube released. **b**, (i) Photo of the SMP adhesive fibril (*R* = 2.5 mm, *h* = 10 mm) with a backing plate (*D* = 25 mm, *t* = 2 mm). The SMP sample is dyed blue for better visualization, and (ii-v) photos of the SMP adhesive holding glass bottles with different volumes filled with water (ii, 100 ml, 292 g; iii, 250 ml, 535 g; iv, 500 ml, 970 g; vi, 1000 ml, 1660 g).

Section S12. Monitoring of the heating process of the SMP fibrillar adhesives

The adhesive hook utilizing SMP fibrillar adhesive as a superglue can be removed easily with the assistance of only a hair dryer. Due to the space in between the SMP adhesive fibrils, the SMP fibril arrays can be heated quickly as compared to the large single fibril adhesive with a comparable apparent contact area. To demonstrate this, a thermal imaging system (FLUKE, Ti200) is used to monitor the temperature of the SMP fibril arrays and the single fibril adhesive with a comparable apparent contact area when they are heated by a hair dryer, as illustrated in Fig. S13. Small holes are opened on the acrylic adherend, which facilitates exposure of the adhesive tip adhering to the adherend and direct monitoring of its temperature. Fig. S14-a shows the temperature evolution at the center of the central fibril of the SMP fibril arrays in contact with the acrylic adherend as compared to that at the center of the large single fibril adhesive when heated by a hair dryer laterally. It can be seen that the SMP fibril arrays are heated much faster than the SMP single fibril adhesive with the comparable apparent contact area. For the fibril arrays, it takes less than one minute to heat the fibril tip to 60 ℃, even with an apparent contact area of 1194.8 mm^2. However, this process takes more than 30 minutes for the single fibril with a comparable apparent contact area of 1017.9 mm^2, as shown in Fig. S14-b.

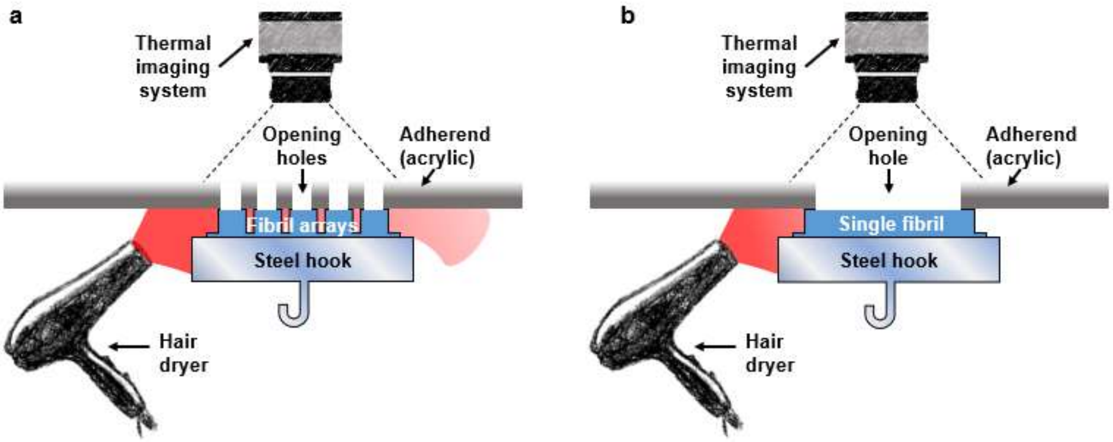

Fig. S13. Illustrations of the set-up used to measure the heating process of the SMP: **a**, fibril arrays and **b**, a single fibril adhesive.

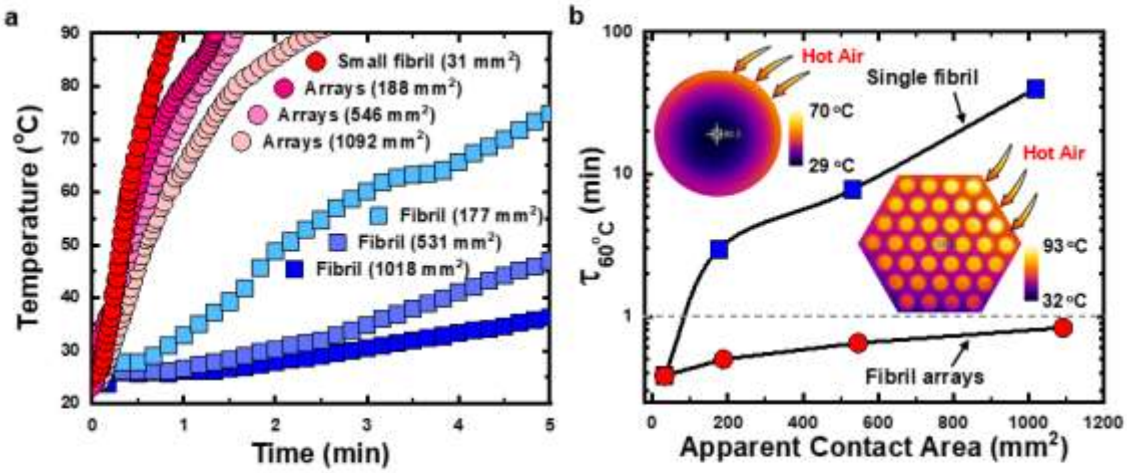

Fig. S14. Heating process of a SMP single fibril adhesive and fibril arrays using a hair dryer. **a**, Temperature evolution at the center of the central fibril of the SMP fibril arrays in contact with the acrylic adherend as compared to that at the center of the large single fibril with comparable apparent contact area when heated by a hair dryer laterally. **b**, Time required to heat the center of the SMP fibril tip in contact with the adherend to 60 ℃ using a hair dryer. Inset shows the temperature distribution of the SMP fibril arrays (apparent contact area 1194.8 mm^2) and the single fibril (apparent contact area 1017.9 mm^2) after heated for 3 minutes. The small fibril refers to a fibril with a radius R = 2.5 mm, the fibril arrays are shown in Fig. S9a and single fibril adhesives in Fig. S9b.

Section S13. Summary and comparison of the performance of different fibrillar adhesive designs.

The scaling limit at fibril (critical fibril radius when adhesion strength is 1 MPa) and array levels (effective array adhesion when the apparent contact area is scaled up to 1000 mm^2), adhesion switchability when the adhesion strength is on the order of 100 kPa, the surface roughness adaptability (RMS roughness value of the adherend surface when adhesion strength is reduced by 50%) and the adhesion durability of the SMP R2G fibrillar adhesive are comprehensively compared with those of the gecko fibrillar adhesives [80, 83, 85, 119-122] and bioinspired fibrillar adhesives with flat [27, 33, 119, 123, 124], mushroom [32, 119, 125, 126], gradient [24, 119, 127, 128], gradient-mushroom [24, 119, 127, 128] designs, as shown in Table S3. From Table S3 we can see that the SMP R2G fibrillar adhesives exhibit superior performance over other designs in all five aspects.

Table S3. Summary and comparison of the performance of different fibrillar adhesive designs

Description	R2G fibril	Flat fibril	Mushroom fibril	Gradient fibril	Gradient mushroom	Gecko
**Critical fibril radius at 1 MPa (μm)** Critical fibril radius when adhesion strength is 1 MPa	> 10 mm Fig. 1c	~ 0.25 μm Fig. S1	~ 10 μm Fig. S1	~ 1 μm Fig. S1	~ 10 μm Fig. S1	~ 0.1 μm Ref [83]
**Array adhesion strength at 10^3 mm^2 (MPa)** Effective adhesion strength when the apparent contact area is scaled up to 1000 mm^2	> 1 MPa Fig. 1e	< 10 kPa Figs. S1, S3	< 50 kPa Figs. S1, S3	<100 kPa Figs. S1, S3	<100 kPa Figs. S1, S3	~ 18 kPa Ref [80]
**Adhesion switchability at 100 kPa** Reported adhesion switchability when the adhesion strength is on the order of 100 kPa	> 1000 Figs. 1d, 1f	~ 200 Ref [119]	~ 30 Ref [119]	~ 10 Ref [119]	< 10 Ref [119]	~ 200 Ref [119]
**Adaptable surface roughness (RMS) value (μm)** RMS roughness value of the adherend surface when adhesion strength is reduced by 50%	>10 μm Ref [119]	~ 0.2 μm Ref [124]	~ 0.2 μm Ref [125, 126]	~ 1 μm Ref [24, 127]	~ 1 μm Ref [24, 127]	~ 1 μm Ref [85, 120-122]
**Adhesion Durability** Repeated cycle before observable fibril collapse, bundling or breaking	>100 Fig. 4b	~ 10 Ref [27, 33, 123]	~ 50 Ref [32]	>100 Ref [27]	>100 Ref [128]	>100 Ref [122]

Supplementary movies

Movie S1. Detachment of an SMP adhesive fibril in the R2G condition and rubber-phase.

Movie S2. Detachment of SMP fibril arrays in the R2G condition and rubber-phase.

Movie S3. Gripping and release of a wood cube using a single SMP fibril adhesive.

Movie S4. Loading demonstrations utilizing the SMP fibril arrays as the superglue for adhesive hooks - Part I.

Movie S5. Loading demonstrations utilizing the SMP fibril arrays as the superglue for adhesive hooks - Part II.

Movie S6. Comparison of the heating process of a single fibril adhesive and fibril arrays.

Movie S7. On-demand detachment of the SMP fibrillar adhesive using a hair dryer

References

1. Sitti M, Fearing RS. Synthetic gecko foot-hair micro/nano-structures as dry adhesives. *Journal of Adhesion Science and Technology*. 2003; **17**(8): 1055-1073.
2. Wang X, Tan D, Zhang X *et al.* Effective elastic modulus of structured adhesives: from biology to biomimetics. *Biomimetics*. 2017; **2**(3): 10.
3. Jagota A, Hui C-Y. Adhesion, friction, and compliance of bio-mimetic and bio-inspired structured interfaces. *Materials Science and Engineering: R: Reports*. 2011; **72**(12): 253-292.
4. Gao H, Wang X, Yao H *et al.* Mechanics of hierarchical adhesion structures of geckos. *Mechanics of Materials*. 2005; **37**(2): 275-285.
5. Yao H, Gao H. Optimal shapes for adhesive binding between two elastic bodies. *Journal of Colloid and Interface Science*. 2006; **298**(2): 564-572.
6. Carbone G, Pierro E, Gorb SN. Origin of the superior adhesive performance of mushroom-shaped microstructured surfaces. *Soft Matter*. 2011; **7**(12): 5545-5552.
7. Hensel R, Moh K, Arzt E. Engineering micropatterned dry adhesives: from contact theory to handling applications. *Advanced Functional Materials*. 2018; **28**(28): 1800865.
8. Purtov J, Frensemeier M, Kroner E. Switchable adhesion in vacuum using bio-inspired dry adhesives. *ACS Applied Materials & Interfaces*. 2015; **7**(43): 24127-24135.
9. Bauer CT, Kroner E, Fleck NA *et al.* Hierarchical macroscopic fibrillar adhesives: in situ study of buckling and adhesion mechanisms on wavy substrates. *Bioinspiration & Biomimetics*. 2015; **10**(6): 066002.
10. Barreau V, Hensel R, Guimard NK *et al.* Fibrillar elastomeric micropatterns create tunable adhesion even to rough surfaces. *Advanced Functional Materials*. 2016; **26**(26): 4687-4694.
11. Song S, Drotlef D-M, Majidi C *et al.* Controllable load sharing for soft adhesive interfaces on three-dimensional surfaces. *Proceedings of the National Academy of Sciences*. 2017; **114**(22): E4344-E4353.
12. Tinnemann V, Arzt E, Hensel R. Switchable double-sided micropatterned adhesives for selective fixation and detachment. *Journal of the Mechanics and Physics of Solids*. 2019; **123**: 20-27.
13. Lee SH, Hwang I, Kang BS *et al.* Highly flexible and self-adaptive dry adhesive end-effectors for precision robotics. *Soft Matter*. 2019; **15**(29): 5827-5834.
14. Mohammadi Nasab A, Luo A, Sharifi S *et al.* Switchable adhesion via subsurface pressure modulation. *ACS Applied Materials & Interfaces*. 2020; **12**(24): 27717-27725.
15. van Assenbergh P, Zhang K, Buijnsters JG *et al.* Effect of lateral reinforcements on the adhesion and friction of micropillar adhesives. *Applied Physics A*. 2020; **126**(10): 1-9.
16. Liu Q, Tan D, Meng F *et al.* Adhesion enhancement of micropillar array by combining the adhesive design from gecko and tree frog. *Small*. 2021; **17**(4): 2005493.
17. Yang Y, Xu T, Bei HP *et al.* Sculpting bio-inspired surface textures: an adhesive janus periosteum. *Advanced Functional Materials*. 2021; **31**(37): 2104636.
18. Wang Y, Zhang X, Hensel R *et al.* Sliding mechanism for release of superlight objects from micropatterned adhesives. *Advanced Materials Interfaces*. 2022; **9**(5): 2101764.
19. Zhao J, Li X, Tan Y *et al.* Smart adhesives via magnetic actuation. *Advanced Materials*. 2022; **34**(8): 2107748.
20. Hu H, Wang D, Tian H *et al.* Bioinspired hierarchical structures for contact-sensible adhesives. *Advanced Functional Materials*. 2022; **32**(8): 2109076.
21. Murphy MP, Aksak B, Sitti M. Gecko-inspired directional and controllable adhesion. *Small*. 2009; **5**(2): 170-175.
22. Jin K, Cremaldi JC, Erickson JS *et al.* Biomimetic bidirectional switchable adhesive inspired by the gecko. *Advanced Functional Materials*. 2014; **24**(5): 574-579.
23. Mengüç Y, Yang SY, Kim S *et al.* Gecko-inspired controllable adhesive structures applied to micromanipulation. *Advanced Functional Materials*. 2012; **22**(6): 1246-1254.
24. Gorumlu S, Aksak B. Sticking to rough surfaces using functionally graded bio-inspired microfibres. *Royal Society Open Science*. 2017; **4**(6): 161105.
25. Fischer SCL, Arzt E, Hensel R. Composite pillars with a tunable interface for adhesion to rough substrates. *ACS Applied Materials & Interfaces*. 2017; **9**(1): 1036-1044.

26. Jeong HE, Lee J-K, Kim HN *et al.* A nontransferring dry adhesive with hierarchical polymer nanohairs. *Proceedings of the National Academy of Sciences*. 2009; **106**(14): 5639-5644.
27. Wang Z. Slanted functional gradient micropillars for optimal bioinspired dry adhesion. *ACS nano*. 2018; **12**(2): 1273-1284.
28. Seong M, Hwang I, Park S *et al.* Enhanced thermal transport across self-interfacing van der waals contacts in flexible thermal devices. *Advanced Functional Materials*. 2021; **31**(48): 2107023.
29. Kim Y, Yang C, Kim Y *et al.* Designing an adhesive pillar shape with deep learning-based optimization. *ACS Applied Materials & Interfaces*. 2020; **12**(21): 24458-24465.
30. Son D, Liimatainen V, Sitti M. Machine learning‐based and experimentally validated optimal adhesive fibril designs. *Small*. 2021; **17**(39): 2102867.
31. Luo A, Zhang H, Turner KT. Machine learning-based optimization of the design of composite pillars for dry adhesives. *Extreme Mechanics Letters*. 2022; **54**: 101695.
32. Zhao J, Lu T, Pan T *et al.* Mushroom-shaped micropillar with a maximum pull-off force. *Journal of Applied Mechanics*. 2022; **89**(7).
33. Geim AK, Dubonos S, Grigorieva I *et al.* Microfabricated adhesive mimicking gecko foot-hair. *Nature Materials*. 2003; **2**(7): 461-463.
34. Zhao Y, Tong T, Delzeit L *et al.* Interfacial energy and strength of multiwalled-carbon-nanotube-based dry adhesive. *Journal of Vacuum Science & Technology B: Microelectronics and Nanometer Structures Processing, Measurement, and Phenomena*. 2006; **24**(1): 331-335.
35. Lee H, Lee BP, Messersmith PB. A reversible wet/dry adhesive inspired by mussels and geckos. *Nature*. 2007; **448**(7151): 338-341.
36. Reddy S, Arzt E, del Campo A. Bioinspired surfaces with switchable adhesion. *Advanced materials*. 2007; **19**(22): 3833-3837.
37. Qu L, Dai L, Stone M *et al.* Carbon nanotube arrays with strong shear binding-on and easy normal lifting-off. *Science*. 2008; **322**(5899): 238-242.
38. Boesel LF, Greiner C, Arzt E *et al.* Gecko‐inspired surfaces: a path to strong and reversible dry adhesives. *Advanced Materials*. 2010; **22**(19): 2125-2137.
39. Bhushan B, Lee H. Fabrication and characterization of multi-level hierarchical surfaces. *Faraday Discussions*. 2012; **156**(1): 235-241.
40. Palacio ML, Bhushan B, Schricker SR. Gecko-inspired fibril nanostructures for reversible adhesion in biomedical applications. *Materials Letters*. 2013; **92**: 409-412.
41. Izadi H, Golmakani M, Penlidis A. Enhanced adhesion and friction by electrostatic interactions of double-level Teflon nanopillars. *Soft Matter*. 2013; **9**(6): 1985-1996.
42. Zhang H, Wu L, Jia S *et al.* Fabrication and adhesion of hierarchical micro-seta. *Chinese Science Bulletin*. 2012; **57**(11): 1343-1349.
43. Minsky H, Turner K. Achieving enhanced and tunable adhesion via composite posts. *Applied Physics Letters*. 2015; **106**(20): 201604.
44. Luo A, Nasab AM, Tatari M *et al.* Adhesion of flat-ended pillars with non-circular contacts. *Soft Matter*. 2020; **16**(41): 9534-9542.
45. Peisker H, Michels J, Gorb SN. Evidence for a material gradient in the adhesive tarsal setae of the ladybird beetle Coccinella septempunctata. *Nature Communications*. 2013; **4**(1): 1-7.
46. Dong X, Zhang R, Tian Y *et al.* Functionally graded gecko setae and the biomimics with robust adhesion and durability. *ACS Applied Polymer Materials*. 2020; **2**(7): 2658-2666.
47. Murphy MP, Kim S, Sitti M. Enhanced adhesion by gecko-inspired hierarchical fibrillar adhesives. *ACS Applied Materials & Interfaces*. 2009; **1**(4): 849-855.
48. Röhrig M, Thiel M, Worgull M *et al.* 3D direct laser writing of nano-and microstructured hierarchical gecko-mimicking surfaces. *Small*. 2012; **8**(19): 3009-3015.
49. Baik S, Lee J, Jeon EJ *et al.* Diving beetle–like miniaturized plungers with reversible, rapid biofluid capturing for machine learning–based care of skin disease. *Science Advances*. 2021; **7**(25): eabf5695.
50. Song S, Sitti M. Soft grippers using micro‐fibrillar adhesives for transfer printing. *Advanced Materials*. 2014; **26**(28): 4901-4906.

51. Lee H, Um DS, Lee Y *et al.* Octopus‐inspired smart adhesive pads for transfer printing of semiconducting nanomembranes. *Advanced Materials*. 2016; **28**(34): 7457-7465.
52. Bae W-G, Kim D, Suh K-Y. Instantly switchable adhesion of bridged fibrillar adhesive via gecko-inspired detachment mechanism and its application to a transportation system. *Nanoscale*. 2013; **5**(23): 11876-11884.
53. Yoo B, Cho S, Seo S *et al.* Elastomeric angled microflaps with reversible adhesion for transfer-printing semiconductor membranes onto dry surfaces. *ACS Applied Materials & Interfaces*. 2014; **6**(21): 19247-19253.
54. Seo S, Lee J, Kim K-S *et al.* Anisotropic adhesion of micropillars with spatula pads. *ACS Applied Materials & Interfaces*. 2014; **6**(3): 1345-1350.
55. Linghu C, Wang C, Cen N *et al.* Rapidly tunable and highly reversible bio-inspired dry adhesion for transfer printing in air and a vacuum. *Soft Matter*. 2019; **15**(1): 30-37.
56. Yi H, Seong M, Sun K *et al.* Wet‐responsive, reconfigurable, and biocompatible hydrogel adhesive films for transfer printing of nanomembranes. *Advanced Functional Materials*. 2018; **28**(18): 1706498.
57. Song S, Majidi C, Sitti M. Geckogripper: A soft, inflatable robotic gripper using gecko-inspired elastomer micro-fiber adhesives. In: *2014 IEEE/RSJ International Conference on Intelligent Robots and Systems, 2014*, p. 4624-4629. IEEE.
58. Tian H, Li X, Shao J *et al.* Gecko‐effect inspired soft gripper with high and switchable adhesion for rough surfaces. *Advanced Materials Interfaces*. 2019; **6**(18): 1900875.
59. Shi Z, Tan D, Wang Z *et al.* Switchable adhesion on curved surfaces mimicking the coordination of radial-oriented spatular tips and motion of gecko toes. *ACS Applied Materials & Interfaces*. 2022; **14**(27): 31448-31454.
60. Kim S, Carlson A, Cheng H *et al.* Enhanced adhesion with pedestal-shaped elastomeric stamps for transfer printing. *Applied Physics Letters*. 2012; **100**(17): 171909.
61. Mengüç Y, Yang SY, Kim S *et al.* Gecko‐inspired controllable adhesive structures applied to micromanipulation. *Advanced Functional Materials*. 2012; **22**(6): 1246-1254.
62. Kang SM, Kim JH, Kim SM. Partial wrinkle generation for switchable attachment and high adhesion hysteresis. *International Journal of Precision Engineering and Manufacturing*. 2017; **18**(1): 133-137.
63. Wang Y, Tian H, Shao J *et al.* Switchable dry adhesion with step-like micropillars and controllable interfacial contact. *ACS Applied Materials & Interfaces*. 2016; **8**(15): 10029-10037.
64. Geikowsky E, Aksak B. Bioinspired fibrillar adhesives with shape-controlled off-center caps for switchable and directional adhesion. *Bioinspiration & Biomimetics*. 2020; **15**(5): 056007.
65. Busche JF, Starke G, Knickmeier S *et al.* Controllable dry adhesion based on two-photon polymerization and replication molding for space debris removal. *Micro and Nano Engineering*. 2020; **7**: 100052.
66. Pang H, Pei L, Xu J *et al.* Magnetically tunable adhesion of composite pads with magnetorheological polymer gel cores. *Composites Science and Technology*. 2020; **192**: 108115.
67. Ye Z, Lum GZ, Song S *et al.* Phase change of gallium enables highly reversible and switchable adhesion. *Advanced Materials*. 2016; **28**(25): 5088-5092.
68. Krahn J, Sameoto D, Menon C. Controllable biomimetic adhesion using embedded phase change material. *Smart Materials and Structures*. 2010; **20**(1): 015014.
69. Qu L, Dai L. Gecko‐foot‐mimetic aligned single‐walled carbon nanotube dry adhesives with unique electrical and thermal properties. *Advanced materials*. 2007; **19**(22): 3844-3849.
70. Li C, Ouyang L, Armstrong JP *et al.* Advances in the fabrication of biomaterials for gradient tissue engineering. *Trends in Biotechnology*. 2021; **39**(2): 150-164.
71. Stork NE. A scanning electron microscope study of tarsal adhesive setae in the Coleoptera. 1980.
72. Walker G, Yulf A, Ratcliffe J. The adhesive organ of the blowfly, Calliphora vomitoria: a functional approach (Diptera: Calliphoridae). *Journal of Zoology*. 1985; **205**(2): 297-307.
73. Lees A, Hardie J. The organs of adhesion in the aphid Megoura viciae. *Journal of Experimental Biology*. 1988; **136**(1): 209-228.
74. Walker G. Adhesion to smooth surfaces by insects—a review. *International Journal of Adhesion and Adhesives*. 1993; **13**(1): 3-7.
75. Kesel A, Martin A, Seidl T. Adhesion measurements on the attachment devices of the jumping spider Evarcha arcuata. *Journal of Experimental Biology*. 2003; **206**(16): 2733-2738.

76. Langer MG, Ruppersberg JP, Gorb S. Adhesion forces measured at the level of a terminal plate of the fly's seta. *Proceedings of the Royal Society of London Series B: Biological Sciences*. 2004; **271**(1554): 2209-2215.
77. Frantsevich L, Ji A, Dai Z *et al.* Adhesive properties of the arolium of a lantern-fly, Lycorma delicatula (Auchenorrhyncha, Fulgoridae). *Journal of insect physiology*. 2008; **54**(5): 818-827.
78. Al Bitar L, Voigt D, Zebitz CP *et al.* Tarsal morphology and attachment ability of the codling moth Cydia pomonella L.(Lepidoptera, Tortricidae) to smooth surfaces. *Journal of Insect Physiology*. 2009; **55**(11): 1029-1038.
79. Labonte D, Federle W. Functionally different pads on the same foot allow control of attachment: stick insects have load-sensitive "heel" pads for friction and shear-sensitive "toe" pads for adhesion. *PLoS One*. 2013; **8**(12): e81943.
80. Tao D, Wan J, Pesika NS *et al.* Adhesion and friction of an isolated gecko setal array: The effects of substrates and relative humidity. *Biosurface and Biotribology*. 2015; **1**(1): 42-49.
81. Voigt D, Tsipenyuk A, Varenberg M. How tight are beetle hugs? Attachment in mating leaf beetles. *Royal Society open science*. 2017; **4**(9): 171108.
82. Autumn K, Sitti M, Liang YA *et al.* Evidence for van der Waals adhesion in gecko setae. *Proceedings of the National Academy of Sciences*. 2002; **99**(19): 12252-12256.
83. Gao H, Yao H. Shape insensitive optimal adhesion of nanoscale fibrillar structures. *Proceedings of the National Academy of Sciences*. 2004; **101**(21): 7851-7856.
84. Irschick DJ, Austin CC, Petren K *et al.* A comparative analysis of clinging ability among pad-bearing lizards. *Biological journal of the Linnean Society*. 1996; **59**(1): 21-35.
85. Autumn K, Liang YA, Hsieh ST *et al.* Adhesive force of a single gecko foot-hair. *Nature*. 2000; **405**(6787): 681-685. doi: 10.1038/35015073
86. Yu D, Hensel R, Beckelmann D *et al.* Tailored polyurethane acrylate blend for large-scale and high-performance micropatterned dry adhesives. *Journal of Materials Science*. 2019; **54**(19): 12925-12937.
87. Zhang T, Yuk H, Lin S *et al.* Tough and tunable adhesion of hydrogels: experiments and models. *Acta Mechanica Sinica*. 2017; **33**(3): 543-554.
88. Cho H, Wu G, Jolly JC *et al.* Intrinsically reversible superglues via shape adaptation inspired by snail epiphragm. *Proceedings of the National Academy of Sciences*. 2019; **116**(28): 13774-13779.
89. Fan H, Wang J, Gong JP. Barnacle cement proteins‐inspired tough hydrogels with robust, long‐lasting, and repeatable underwater adhesion. *Advanced Functional Materials*. 2021; **31**(11): 2009334.
90. Eisenhaure JD, Xie T, Varghese S *et al.* Microstructured shape memory polymer surfaces with reversible dry adhesion. *ACS Applied Materials & Interfaces*. 2013; **5**(16): 7714-7717.
91. Eisenhaure JD, Rhee SI, Ala'a M *et al.* The use of shape memory polymers for microassembly by transfer printing. *Journal of Microelectromechanical Systems*. 2014; **23**(5): 1012-1014.
92. Moslemim M, Khoshravan M. Cohesive zone parameters selection for mode-I prediction of interfacial delamination/Izbira parametrov kohezijske cone za napovedovanje medpovrsinske delaminacije tipa I. *Strojniski Vestnik-Journal of Mechanical Engineering*. 2015; **61**(9): 507-517.
93. Zheng N, Fang G, Cao Z *et al.* High strain epoxy shape memory polymer. *Polymer Chemistry*. 2015; **6**(16): 3046-3053.
94. Huang Y, Zheng N, Cheng Z *et al.* Direct laser writing-based programmable transfer printing via bioinspired shape memory reversible adhesive. *ACS Applied Materials & Interfaces*. 2016; **8**(51): 35628-35633.
95. Park JK, Eisenhaure JD, Kim S. Reversible underwater dry adhesion of a shape memory polymer. *Advanced Materials Interfaces*. 2019; **6**(3): 1801542.
96. Linghu C, Zhang S, Wang C *et al.* Universal SMP gripper with massive and selective capabilities for multiscaled, arbitrarily shaped objects. *Science advances*. 2020; **6**(7): eaay5120.
97. Pina-Hernandez C, Kim JS, Guo LJ *et al.* High-throughput and etch-selective nanoimprinting and stamping based on fast-thermal-curing poly(dimethylsiloxane)s. *Advanced materials*. 2007; **19**(9): 1222-1227.
98. Liu M, Sun J, Chen Q. Influences of heating temperature on mechanical properties of polydimethylsiloxane. *Sensors and Actuators A: Physical*. 2009; **151**(1): 42-45.
99. Jeong SH, Zhang S, Hjort K *et al.* PDMS‐based elastomer tuned soft, stretchable, and sticky for epidermal electronics. *Advanced Materials*. 2016; **28**(28): 5830-5836.
100. Cai M, Nie S, Du Y *et al.* Soft elastomers with programmable stiffness as strain-isolating substrates for stretchable electronics. *ACS Applied Materials & Interfaces*. 2019; **11**(15): 14340-14346.

101. Meitl MA, Zhu Z-T, Kumar V *et al.* Transfer printing by kinetic control of adhesion to an elastomeric stamp. *Nature Materials*. 2006; **5**(1): 33-38.
102. Feng X, Meitl MA, Bowen AM *et al.* Competing fracture in kinetically controlled transfer printing. *Langmuir*. 2007; **23**(25): 12555-12560.
103. Chen H, Feng X, Huang Y *et al.* Experiments and viscoelastic analysis of peel test with patterned strips for applications to transfer printing. *Journal of the Mechanics and Physics of Solids*. 2013; **61**(8): 1737-1752.
104. Tiwari A, Dorogin L, Bennett A *et al.* The effect of surface roughness and viscoelasticity on rubber adhesion. *Soft matter*. 2017; **13**(19): 3602-3621.
105. Peng P, Wu K, Lv L *et al.* One-step selective adhesive transfer printing for scalable fabrication of stretchable electronics. *Advanced Materials Technologies*. 2018; **3**(3): 1700264.
106. Santos D, Spenko M, Parness A *et al.* Directional adhesion for climbing: theoretical and practical considerations. *Journal of Adhesion Science and Technology*. 2007; **21**(12-13): 1317-1341.
107. Jiang C, Zhang L, Yang Q *et al.* Self-healing polyurethane-elastomer with mechanical tunability for multiple biomedical applications in vivo. *Nature Communications*. 2021; **12**(1): 1-13.
108. Mengüç Y, Röhrig M, Abusomwan U *et al.* Staying sticky: contact self-cleaning of gecko-inspired adhesives. *Journal of The Royal Society Interface*. 2014; **11**(94): 20131205.
109. Cui W, Zhu R, Zheng Y *et al.* Transforming non-adhesive hydrogels to reversible tough adhesives via mixed-solvent-induced phase separation. *Journal of Materials Chemistry A*. 2021; **9**(15): 9706-9718.
110. Zhao X, Peng L-M, Chen Y *et al.* Phase change mediated mechanically transformative dynamic gel for intelligent control of versatile devices. *Materials Horizons*. 2021; **8**(4): 1230-1241.
111. Yang J, Bai R, Chen B *et al.* Hydrogel adhesion: a supramolecular synergy of chemistry, topology, and mechanics. *Advanced Functional Materials*. 2020; **30**(2): 1901693.
112. Xue Y, Zhang J, Chen X *et al.* Trigger‐detachable hydrogel adhesives for bioelectronic interfaces. *Advanced Functional Materials*. 2021; **31**(47): 2106446.
113. Swadener J, Liechti K, De Lozanne A. The intrinsic toughness and adhesion mechanisms of a glass/epoxy interface. *Journal of the Mechanics and Physics of Solids*. 1999; **47**(2): 223-258.
114. Xu H, Zhang X, Hu G *et al.* A special filler for epoxy resin to enhance the T peel strength of adhesive. *Polymer Composites*. 2020; **41**(10): 4372-4378.
115. Ogden RW, Roxburgh DG. A pseudo–elastic model for the Mullins effect in filled rubber. *Proceedings of the Royal Society of London Series A: Mathematical, Physical and Engineering Sciences*. 1999; **455**(1988): 2861-2877.
116. Peng B, Feng X-Q, Li Q. Decohesion of a rigid flat punch from an elastic layer of finite thickness. *Journal of the Mechanics and Physics of Solids*. 2020; **139**: 103937.
117. Arzt E, Quan H, McMeeking RM *et al.* Functional surface microstructures inspired by nature – From adhesion and wetting principles to sustainable new devices. *Progress in Materials Science*. 2021; **120**: 100823.
118. Wu J, Guo J, Linghu C *et al.* Rapid digital light 3D printing enabled by a soft and deformable hydrogel separation interface. *Nature Communications*. 2021; **12**(1): 1-9.
119. Linghu C, Liu Y, Tan YY *et al.* Overcoming the adhesion paradox and switchability conflict on rough surfaces with shape-memory polymers. *Proceedings of the National Academy of Sciences*. 2023; **120**(13): e2221049120.
120. Huber G, Gorb SN, Hosoda N *et al.* Influence of surface roughness on gecko adhesion. *Acta biomaterialia*. 2007; **3**(4): 607-610.
121. Autumn K, Majidi C, Groff R *et al.* Effective elastic modulus of isolated gecko setal arrays. *Journal of Experimental Biology*. 2006; **209**(18): 3558-3568.
122. Autumn K. Gecko adhesion: structure, function, and applications. *MRS bulletin*. 2007; **32**(6): 473-478.
123. Suthisomboon T, Rukpanich T, Asawalertsak N *et al.* VENOM: Versatile, adhesive, and soft material for various surface adhesion. In: *2021 IEEE 4th International Conference on Soft Robotics (RoboSoft), 2021*, p. 543-546. IEEE.
124. Kim J-K, Varenberg M. Contact splitting in dry adhesion and friction: reducing the influence of roughness. *Beilstein Journal of Nanotechnology*. 2019; **10**: 1-8.
125. Cañas N, Kamperman M, Völker B *et al.* Effect of nano- and micro-roughness on adhesion of bioinspired micropatterned surfaces. *Acta Biomaterialia*. 2012; **8**(1): 282-288.
126. Kasem H, Varenberg M. Effect of counterface roughness on adhesion of mushroom-shaped microstructure. *Journal of The Royal Society Interface*. 2013; **10**(87): 20130620.
127. Moreira Lana G, Zhang X, Müller C *et al.* Film-terminated fibrillar microstructures with improved adhesion on skin-like surfaces. *ACS Appl Mater Interfaces*. 2022; **14**(41): 46239-46251.

128. Drotlef DM, Amjadi M, Yunusa M *et al.* Bioinspired composite microfibers for skin adhesion and signal amplification of wearable sensors. *Advanced materials*. 2017; **29**(28): 1701353.
